# Multicomponent Synthesis of Multi-Target Quinazolines Modulating Cholinesterase, Oxidative Stress, and Amyloid Aggregation Activities for the Therapy of Alzheimer’s Disease

**DOI:** 10.3390/molecules30193930

**Published:** 2025-09-30

**Authors:** Saida Chakhari, José Marco-Contelles, Isabel Iriepa, Maria do Carmo Carreiras, Fakher Chabchoub, Lhassane Ismaili, Bernard Refouvelet

**Affiliations:** 1 Laboratory of Applied Chemistry: Heterocycles, Lipids and Polymers, Faculty of Sciences of Sfax, University of Sfax, Sfax 3000, Tunisia; saida.chakhari@univ-fcomte.fr; 2 INSERM UMR1322 LINC, Université Marie et Louis Pasteur, F-25000 Besançon, France; bernard.refouvelet@univ-fcomte.fr; 3 Laboratory of Medicinal Chemistry (IQOG, CSIC) C/Juan de la Cierva 3, 28006 Madrid, Spain; jlmarco@iqog.csic.es; 4 Departamento de Química Orgánica y Química Inorgánica, Universidad de Alcalá, 28805 Alcalá de Henares, Spain; isabel.iriepa@uah.es; 5 Instituto de Investigación Química “Andrés M. del Río” (IQAR), 28805 Alcalá de Henares, Spain; 6 Research Institute for Medicines (iMed.ULisboa), Faculty of Pharmacy, Universidade de Lisboa, 1649-003 Lisboa, Portugal; maria.carreiras@gmail.com

**Keywords:** antioxidants, cholinesterase inhibitors, ORAC, quinazoline

## Abstract

Alzheimer’s disease (AD) is a multifactorial neurodegenerative disorder characterized by extracellular accumulation of amyloid-beta (Aβ) peptide, intracellular neurofibrillary tangles (NFTs), severe neuronal loss, and a marked decline in cholinergic function. Due to the limited efficacy of currently available therapies, the search for new chemical scaffolds able to target multiple pathological mechanisms remains an urgent priority. Among the most promising strategies are heterocyclic frameworks that can simultaneously interact with cholinesterase (ChE) enzymes and inhibit amyloid-β (Aβ) aggregation while also exhibiting antioxidant activity. In this context, we report a series of quinazoline derivatives synthesized via a sequential, one-pot multicomponent reaction, in good yields. Several of these compounds demonstrated notable antioxidant properties, as well as inhibitory effects on ChE activity and Aβ_1-42_ self-aggregation, highlighting their potential as multifunctional agents for the treatment of neurodegenerative disorders. Notably, 2-ethyl-4-(3,4-Dimethoxyphenyl)aminoquinazoline (**3h**) demonstrated the most balanced biological profile among the tested compounds, exhibiting an ORAC value of **5.73 TE**, an acetylcholinesterase (AChE) inhibition IC_50_ = 6.67 μM, and 36.68% inhibition of Aβ_1–42_ aggregation, closely approaching the activity of curcumin. These findings highlight compound **3h** as a promising quinazoline-based hit for the development of multifunctional agents targeting AD.

## 1. Introduction

Alzheimer’s disease (AD) is a complex, multifactorial neurodegenerative disorder, often associated with complications such as septicemia, infected pressure ulcers, or stroke [1]. AD is primarily characterized by two pathological hallmarks: the extracellular accumulation of amyloid-β (Aβ) peptides and the intracellular formation of neurofibrillary tangles (NFTs), both of which contribute to extensive neuronal loss and reduced choline acetyltransferase activity [2]. In addition to these features, oxidative stress and dysregulation of essential biometal homeostasis, including copper, iron, and zinc, play significant roles in the progression of AD [3,4,5].

The current treatment of AD remains limited, relying mainly on cholinergic and glutamatergic modulation through acetylcholinesterase (AChE) inhibitors, such as donepezil [6], galantamine [7], and rivastigmine [6], as well as *N*-methyl-D-aspartate receptor antagonists, such as memantine [6,8]. Despite their widespread use, these agents provide only modest symptomatic relief without halting disease progression. Consequently, drug discovery efforts have shifted toward multitarget-directed ligands (MTDLs), which can simultaneously interact with various enzymes or receptors implicated in AD pathology [9,10,11,12,13].

Guided by this strategy, our group has previously developed several MTDL candidates [14,15,16] based on nitrogen-containing heterocycles, a versatile class of scaffolds that has been extensively investigated for the design of novel AD therapeutics [17,18,19]. Among the heterocyclic scaffolds explored in AD drug discovery, quinazoline derivatives have emerged as attractive candidates [20]. These bicyclic structures, composed of a fused benzene and pyrimidine ring, exhibit a broad spectrum of biological activities, including neuroprotective, antioxidant, and anti-inflammatory effects [21,22,23]. Several quinazoline-based molecules have been reported to inhibit key enzymes such as AChE and butyrylcholinesterase (BuChE), while also modulating Aβ aggregation pathways, making them promising MTDLs for AD therapy [24,25]. Their structural versatility allows for straightforward chemical modification, facilitating the design of hybrid compounds that can address multiple pathological mechanisms simultaneously. Owing to these properties, quinazolines represent a privileged scaffold for the development of novel bioactive molecules aimed at addressing the multifactorial nature of AD.

Building on our previous work, we report here the synthesis of 17 novel molecules based on the quinazoline scaffold, achieved through an original sequential multicomponent approach designed to combine antioxidant properties with cholinesterase (ChE) inhibition. Biological evaluation revealed that compound **3h** displayed the most balanced profile, with an ORAC value of 5.73 TE, potent AChE inhibition (IC_50_ = 6.67 μM), and 36.68% inhibition of Aβ_1–42_ aggregation. This MTDL thus emerges as a promising hit compound for the development of new quinazoline-based therapeutics for AD.

## 2. Results

### 2.1. Synthesis

**Scheme 1:**
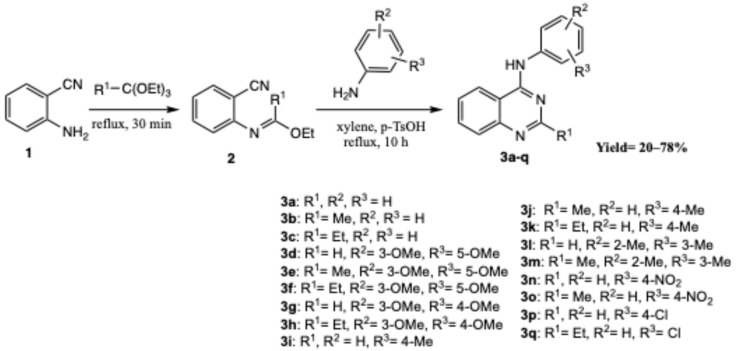
Synthesis of quinazolines **3a**–**q**.

The synthesis of quinazoline derivatives **3a–q** was performed via a sequential, one-pot multicomponent reaction, as illustrated in Scheme 1, affording the desired products in good overall yields. The process begins with the condensation of 2-aminobenzonitrile (1.0 equiv) with the corresponding substituted orthoester (1.2 equiv) under reflux for 30 min, generating an imidate intermediate. After evaporation to remove the excess orthoester and the ethanol formed, the crude intermediate, without isolation, was treated with the appropriate substituted aniline (1.0 equiv) in anhydrous xylene in the presence of a catalytic amount of *p*-toluenesulfonic acid (*p*-TsOH). The reaction mixture was then refluxed for 10 h, yielding quinazolines **3a–q** as precipitated solids.

All synthesized compounds showed analytical and spectroscopic characteristics consistent with their proposed structures (see Materials and Methods and Supplementary Materials; NMR spectra, Figures S1–S21; HRMS spectra, Figures S22–S38).

**Scheme 2:**
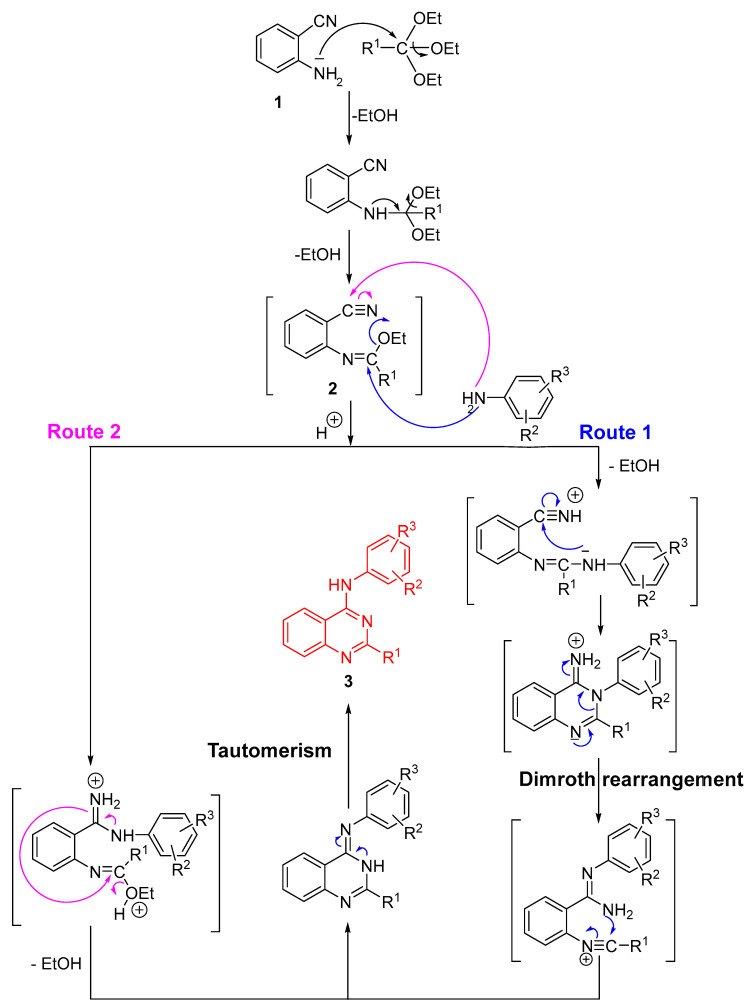
Reaction mechanism for the synthesis of compounds **3a**–**q**.

The synthesis of quinazoline derivatives **3** via a sequential, one-pot process can proceed through two competing mechanistic pathways (Route 1 and Route 2), as depicted in Scheme 2. Initially, 2-aminobenzonitrile (**1**) reacts with the substituted orthoester R^1^-C(OEt)_3_ under acidic conditions to generate an imidate intermediate bearing both a reactive nitrile and an imidoyl group. Protonation enhances nucleophilic activation, thereby enabling subsequent transformation toward the quinazoline scaffold. Upon addition of the substituted aniline, the reaction may proceed through two distinct cyclization routes:

**Route 1:** The aniline nitrogen attacks the electrophilic carbon within the nitrile-imidate system, yielding a cyclized intermediate that undergoes intramolecular rearrangement. This transformation proceeds via a Dimroth rearrangement [26], ultimately leading to quinazoline **3** after tautomerization.

**Route 2:** Alternatively, the aniline nucleophile adds to the imidoyl carbon, producing a protonated intermediate stabilized by intramolecular hydrogen bonding. Subsequent ring closure leads to the same heterocyclic product **3** after tautomerization.

The final tautomeric equilibrium between the imino and amino forms stabilizes the quinazoline structure. These two concurrent mechanistic pathways account for the efficient, high-yielding formation of derivatives **3a**–**q** under reflux conditions in xylene with catalytic p-TsOH.

### 2.2. Biological Evaluation

The newly synthesized compounds were evaluated for their ChE inhibitory activity, antioxidant capacity, and ability to inhibit Aβ_1-42_ self-aggregation.

#### 2.2.1. Inhibition of *Ee*AChE

The inhibitory properties of compounds **3a**–**q** against *Electrophorus electricus* AChE (*Ee*AChE) and equine BuChE (eqBuChE) were assessed using the Ellman colorimetric method [27], with tacrine and donepezil as reference inhibitors [28]. Although the overall potency of these compounds is modest compared with clinically used standards, several derivatives exhibited measurable and selective inhibition, highlighting the potential of this scaffold as a starting point for further optimization.

As summarized in Table 1, compound **3e** emerged as the most promising dual inhibitor, with an IC_50_ value of 6.04 μM for *Ee*AChE and additional activity against eqBuChE (IC_50_ = 9.43 μM). This duality is of particular interest, as BuChE inhibition is considered a valuable therapeutic approach in the later stages of AD [29]. Moreover, the Et-substituted methoxy derivative **3f** and the chloro derivative **3q** displayed selective BuChE inhibition (IC_50_ = 8.50 μM and 8.64 μM, respectively), underscoring the ability of this scaffold to accommodate diverse substituents while retaining measurable activity. By contrast, compound **3h** demonstrated selective *Ee*AChE inhibition (IC_50_ = 6.61 μM) but was inactive against BuChE, indicating that subtle structural variations can modulate enzyme selectivity within this series.

These results allow the derivation of preliminary structure–activity relationships (SAR). Electron-donating methoxy groups located in 3,5- or 3,4-disubstitution patterns, particularly when combined with small alkyl chains (Me or Et), appear to enhance interactions with the ChE active sites. While the absolute potencies are lower than those of tacrine or donepezil, these findings support the quinazoline core as a viable scaffold for the development of selective or dual AChE/BuChE inhibitors, indicating that targeted substituent modifications could significantly enhance affinity.

Overall, the moderate inhibitory activities, combined with the strong antioxidant and anti-amyloid aggregation properties observed in this series (see below), suggest that further structural optimization could yield multifunctional neuroprotective agents, offering promise for addressing the multifactorial nature of AD.

#### 2.2.2. Antioxidant Analysis

The antioxidant capacity of compounds **3a**–**q** was evaluated using the ORAC-FL (Oxygen Radical Absorbance Capacity by Fluorescence) assay, with results expressed as μmol Trolox equivalents (TE) [30,31]. Melatonin, used as a reference compound, displayed an ORAC value of 2.45 TE, consistent with literature data [32]. The ORAC values of the tested series ranged from 0.15 TE (**3c**) to 5.73 TE (**3h**), highlighting a wide variation in radical-scavenging capacity depending on the nature and position of the substituents on the aromatic ring.

Clear SAR was observed. Compounds bearing methoxy substituents exhibited markedly higher antioxidant activity compared with unsubstituted or simply alkyl-substituted derivatives. In particular, the 3,5-dimethoxy derivatives **3d** and **3f** showed ORAC values ranging from 1.29 to 2.85 TE, while the 3,4-dimethoxy derivatives **3g** and **3h** were the most potent of the series, with ORAC values of 5.46 and 5.73 TE, respectively. These findings suggest that electron-donating methoxy groups, especially in the *ortho* and *para* positions, enhance radical stabilization through resonance effects, thereby increasing antioxidant potential. Notably, compounds **3g** and **3h** outperformed both melatonin and ferulic acid (3.7 TE, reported as a standard antioxidant [33]), underscoring the beneficial effect of this substitution pattern. In contrast, derivatives **3a**, **3c**, **3i**, and **3m**, bearing only Me or Et groups, as well as derivatives **3n** and **3q** containing electron-withdrawing substituents such as nitro or chlorine, exhibited weak to moderate antioxidant activities, with ORAC values generally below 1.0 TE. These results indicate that such groups do not significantly contribute to radical stabilization and may even diminish the antioxidant performance by decreasing electron density on the aromatic system.

Overall, these data demonstrate that the presence of multiple methoxy groups, particularly in the 3,4-positions of the aromatic ring, is the key determinant of antioxidant potency within this series. Compounds **3g** and **3h** emerge as the most promising candidates, exhibiting substantially higher radical-scavenging activity than melatonin and even surpassing ferulic acid. These observations highlight the potential of appropriately substituted quinazoline scaffolds for the development of potent antioxidant agents.

#### 2.2.3. Inhibition of Aβ_1–42_ Self- Aggregation

**Figure 1:**
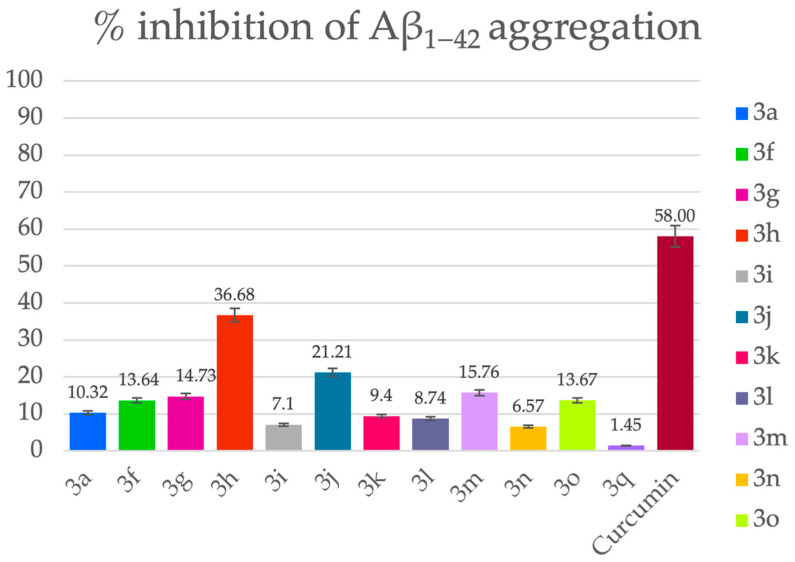
Percentage inhibition of Aβ_1_−_42_ aggregation by the tested compounds. [Aβ] = [Inhibitor] = 50 μM. Data are expressed as mean ± SEM from two independent experiments, each performed in duplicate.

The inhibitory activity of compounds **3a**–**q** against the spontaneous aggregation of Aβ_1–42_ was determined in vitro using a thioflavin T-based fluorometric assay [34] and is summarized in Figure 1. Curcumin was used as a positive control.

Among the tested compounds, **3h** (36.68%) exhibited the most pronounced inhibition, reaching nearly two-thirds of curcumin’s activity. Other methoxy-substituted derivatives, such as **3g** (14.73%) and **3f** (13.64%), also displayed notable effects, suggesting that 3,4- and 3,5-dimethoxy substitution patterns on the aromatic ring contribute favorably to the disruption of Aβ fibrillization. Interestingly, while compound **3f** (R^1^ = Et; R_2_, R_3_ = 3,5-dimethoxy) displayed moderate inhibition, its 3,4-dimethoxy analog **3h** (R_1_ = Et) was substantially more effective, indicating that *para*-orientation of the methoxy groups may enhance binding to Aβ aggregates or interfere with fibril elongation more efficiently.

Beyond the methoxy series, several alkyl- and halogen-substituted derivatives demonstrated measurable inhibition. Notably, **3j** (R^1^ = Me, R^2^ = H, R_3_ = 4-Me) and **3m** (R^1^ = Me; R^2^, R^3^ = 2,3-dimethyl) achieved inhibition levels of 21.21% and 15.76%, respectively, suggesting that small hydrophobic groups can also contribute positively, possibly by interacting with hydrophobic domains within Aβ fibrils. Halogenated derivative **3q** (R^1^ = Et, R^2^ = H, R^3^ = 4-Cl) showed weak inhibition (1.45%), while **3p** (R^1^ = R^2^ = H, R^3^ = 4-Cl) was inactive, indicating that halogenation alone does not favorably modulate aggregation.

Several compounds, including **3b**, **3d**, **3e**, and **3p**, did not display measurable activity under the assay conditions, suggesting that the absence of both electron-donating groups and hydrophobic substituents results in poor interaction with Aβ aggregates.

Collectively, these findings demonstrate that the quinazoline scaffold is capable of modulating Aβ_1–42_ self-aggregation, with the most promising activity observed for the 3,4-dimethoxy substitution pattern in combination with an ethyl side chain (**3h**), as well as for certain hydrophobic derivatives (**3j** and **3m**). Although none of the compounds matched the potency of curcumin, several derivatives, particularly **3h**, showed meaningful inhibitory effects that warrant further investigation. Optimization of substitution patterns, especially those that enhance *para*-methoxy and hydrophobic contributions, could yield derivatives with stronger anti-aggregation potential.

Based on the overall biological evaluation, several derivatives displayed noteworthy activities, with compound **3h** (R^1^ = Et; R^2^, R^3^ = 3,4-dimethoxy) emerging as the most promising MTDL. This compound exhibited the highest ORAC value (5.73 TE), demonstrated *Ee*AChE inhibition (IC_50_ = 6.61 μM), and showed the strongest inhibition of Aβ_1–42_ aggregation (36.68%), approaching the effect of curcumin. Collectively, these results highlight **3h** as a hit compound for further optimization toward the development of new MTDLs for AD.

#### 2.2.4. Molecular Docking Studies for Compounds **3e** and **3h**

Molecular docking studies were performed to elucidate the binding interactions of compounds **3e** and **3h** with *Ee*AChE and eqBuChE. The 3D enzyme structures corresponding to the biological targets were retrieved from the Protein Data Bank. For *Ee*AChE, the crystal structure (PDB ID: 1C2B) was selected, and docking was carried out on a single catalytic subunit, allowing eight flexible side chains. Calculations were conducted using AutoDock Vina [35], and docking poses were visualized and analyzed with Discovery Studio 2023.

**Figure 2:**
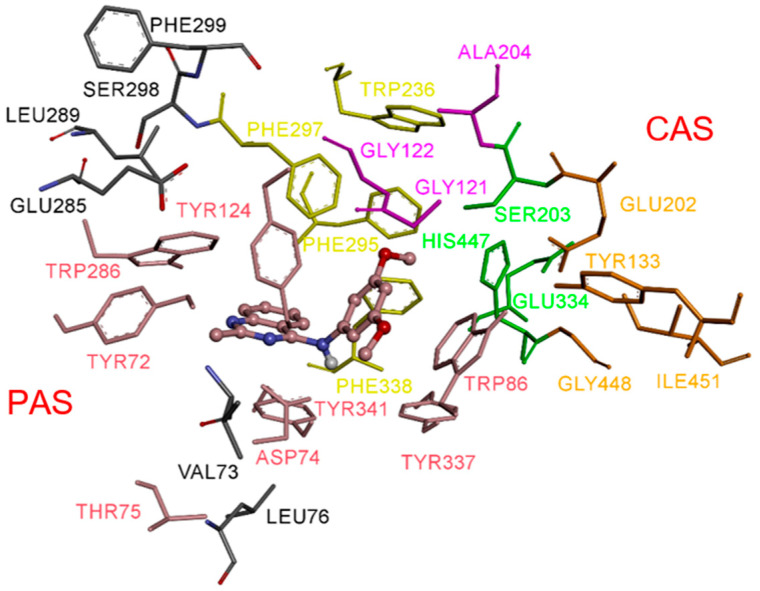
Docked pose of inhibitor **3e** in the active site of *Ee*AChE (binding energy: −10.4 kcal/mol). Compound **3e** is represented as balls and sticks (carbon atoms in brown), and the side-chain conformations of the flexible residues are shown in the same color. The catalytic triad (CT, green), oxyanion hole (OH, magenta), anionic subsite (AS, orange, except Trp86), acyl-binding pocket (ABP, yellow), and peripheral anionic site (PAS, brown) are highlighted.

**Figure 3:**
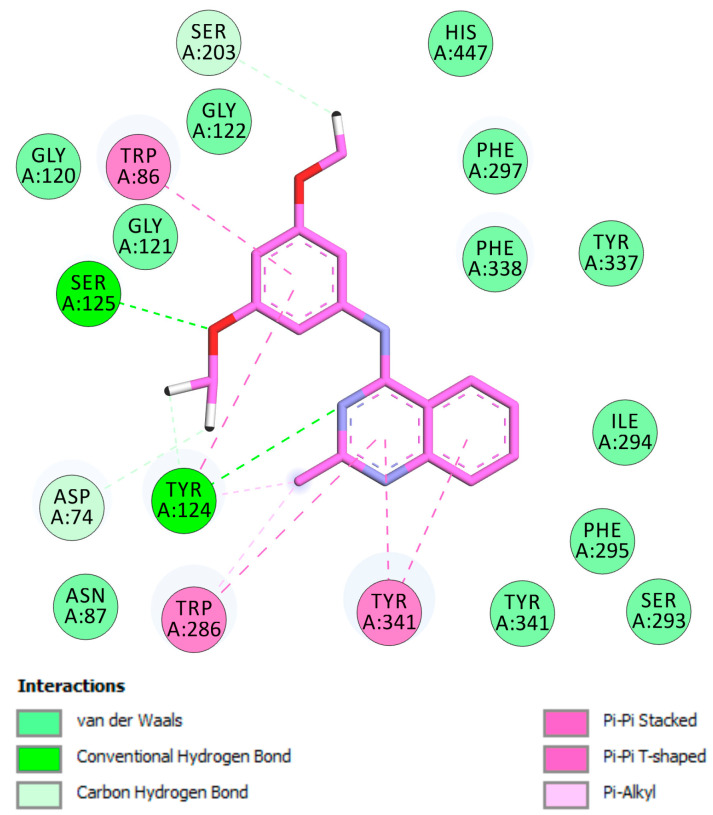
Docked pose of inhibitor **3e** in the active site of *Ee*AChE. Two-dimensional representation of the amino acid residues in the binding site interacting with compound **3e** (pink), including catalytic triad (CT), oxyanion hole (OH), anionic subsite (AS), acyl-binding pocket (ABP), and peripheral anionic site (PAS).

As illustrated in Figure 2, the most energetically favorable pose of compound **3e** occupies the peripheral anionic site (PAS). The quinazoline moiety engages in π–π stacking interactions with Trp286 and Tyr341, while forming a hydrogen bond with Tyr124, contributing to ligand stabilization within the pocket. In this conformation, the methyl substituent is also located in the PAS, interacting with Trp286 and Tyr124 (Figure 3). The dimethoxyphenyl group is oriented toward the catalytic triad residues, reaching the bottom of the active-site gorge, where it establishes stabilizing contacts with Ser203 and His447 (Figure 3), key residues involved in the catalytic mechanism.

**Figure 4:**
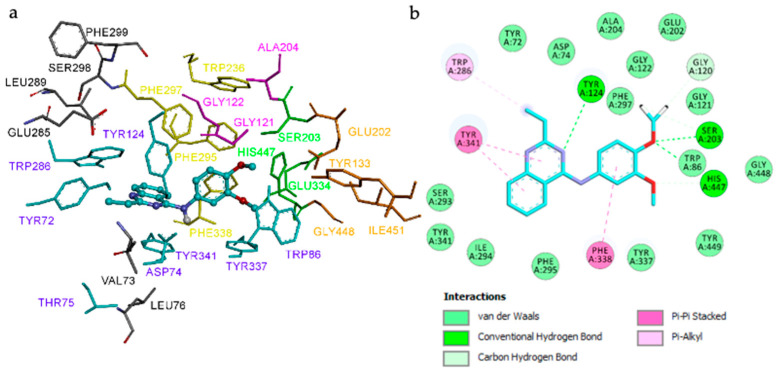
Binding mode of compound **3h** in the active center of *Ee*AChE (binding energy: −10.2 kcal/mol). (**a**) Compound **3h** is represented as balls and sticks (carbon atoms in blue), and the side-chain conformations of the flexible residues are shown in the same color. The catalytic triad (CT, green), oxyanion hole (OH, magenta), anionic subsite (AS, orange, except Trp86), acyl-binding pocket (ABP, yellow), and peripheral anionic site (PAS, blue) are highlighted. (**b**) 2D representation of the amino acid residues in the binding site interacting with compound **3h**.

Compound **3h** adopts a binding orientation within the *Ee*AChE active site, comparable to that observed for **3e**. As shown in Figure 4a, compound **3h** is located at the entire enzymatic gorge, with docking simulations revealing a clear preference for positioning the quinazoline core within the PAS. The dimethoxybenzene moiety is deeply embedded in the catalytic anionic site (CAS), with the amino group lying in the middle of the gorge between CAS and PAS. The dimethoxyphenyl ring is oriented toward the catalytic triad (His447, Ser203, Glu334), forming hydrogen bonds with His447 and Ser203. Within the PAS, the ethyl substituent engages Trp286, and the quinazoline ring establishes π–π stacking interactions with Tyr341 and Tyr124, collectively stabilizing the ligand–enzyme complex (Figure 4b).

To investigate the binding interactions of compounds **3e** and **3h** with eqBuChE, molecular docking studies were performed using a homology model of equine BuChE. Because no X-ray crystal structure is available for eqBuChE, the model was retrieved from the SWISS-MODEL Repository [36] and employed to interpret the experimental data.

**Figure 5:**
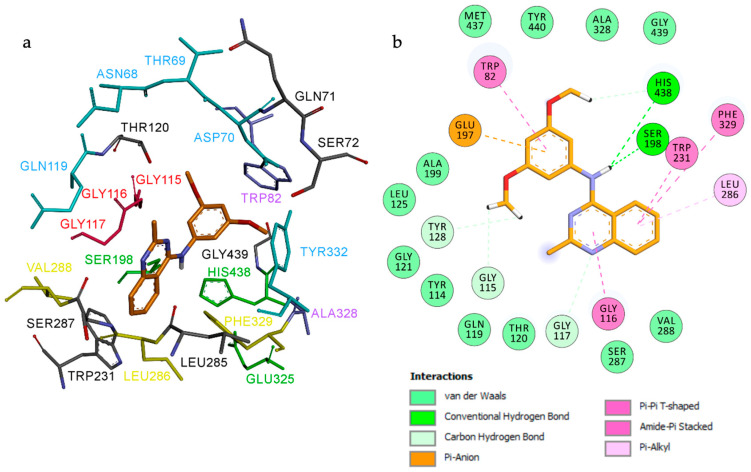
Proposed binding mode for compound **3e** inside the gorge cavity of eqBuChE (binding energy: −8.9 kcal/mol). Compound **3e** is shown in orange. (**a**) Subsites of the active site are colored: catalytic anionic site (CAS, green), oxyanion hole (OH, red), choline-binding site (CBS, violet), acyl-binding pocket (ABP, yellow), and peripheral anionic site (PAS, blue). (**b**) 2D representation of the amino acid residues in the binding site interacting with compound **3e**.

As illustrated in Figure 5, the predicted binding pose of compound **3e** places the quinazoline core in the central region of the receptor cavity, while the dimethoxybenzene moiety penetrates deeply into the gorge (Figure 5a). The amino group is positioned within the catalytic anionic site (CAS), where it forms hydrogen bonds with His438 and Ser198 of the catalytic triad. The quinazoline phenyl ring establishes hydrophobic contacts with Leu286, Trp231, and Phe329 within the acyl-binding pocket (ABP). Additionally, the quinazoline ring interacts with residues of the oxyanion hole (Gly116 and Gly117), and the terminal phenyl ring engages in π–π stacking with Trp82 at the choline-binding site (CBS) (Figure 5b).

**Figure 6:**
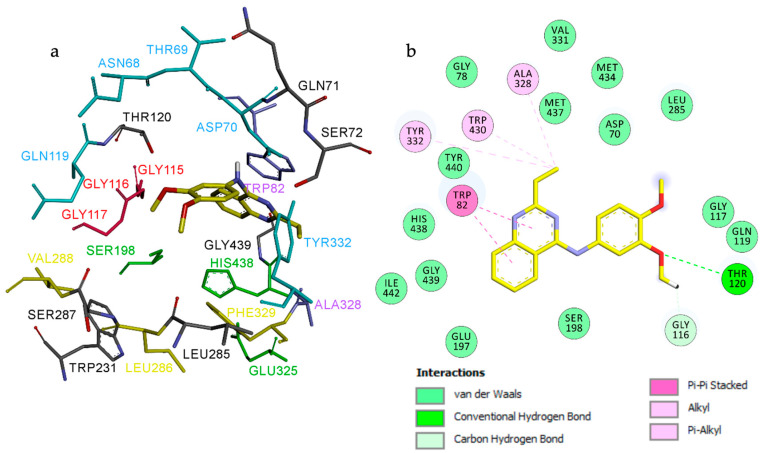
Proposed binding mode for compound **3h** inside the gorge cavity of eqBuChE (binding energy: −8.4 kcal/mol). Compound **3h** is shown in orange. (**a**) Subsites of the active site are colored: catalytic anionic site (CAS, green), oxyanion hole (OH, red), choline-binding site (CBS, violet), acyl-binding pocket (ABP, yellow), and peripheral anionic site (PAS, blue). (**b**) 2D representation of the amino acid residues in the binding site interacting with ligand **3h**.

As illustrated in Figure 6, ligand **3h** occupies the eqBuChE active site and establishes an extensive network of electrostatic and hydrophobic interactions with the receptor. In contrast to **3e**, docking simulations indicate a preferential orientation of the quinazoline core deeper within the active-site cavity, where the dimethylbenzene ring is oriented toward the entrance of the gorge.

The ethyl-quinazoline fragment anchors the ligand through π-π stacking and hydrophobic interactions with Trp82 and Ala328 in the CBS, positioning the ligand away from direct contact with the catalytic triad residues. The ethyl group and dimethylbenzene moiety extend toward the PAS, where they interact with Asp70, Tyr332, and Gln119. Additionally, the ligand engages Gly116 and Gly117 of the oxyanion hole, further stabilizing its binding conformation (Figure 6b).

In conclusion, the molecular docking results indicate that both compounds **3e** and **3h** engage with the CAS and PAS regions of *Ee*AChE, supporting their potential to inhibit Aβ aggregation. Moreover, compound **3e** displayed interactions across multiple subsites of eqBuChE, including the CAS, ABP, OH site, and CBS, suggesting a versatile binding profile. In contrast, compound **3h** showed preferential engagement with the PAS of eqBuChE, reflecting a more selective binding mode. Together, these findings provide valuable structural insights that may guide the rational design and optimization of quinazoline-based multifunctional ligands for the treatment of AD.

#### 2.2.5. Molecular Docking of Compounds **3e** and **3h** into *Ee*AChE and eqBuChE

Compounds **3e** and **3h** were constructed in Discovery Studio 2023 using standard bond lengths and angles. Their geometries were energy-minimized with the adopted-basis Newton-Raphson algorithm, using the CHARMm force field [37] and partial atomic charges. Structures were considered fully optimized when the energy changes between successive iterations fell below 0.001 kcal/mol [38]. Ligands were prepared for docking using AutoDockTools (ADT, v1.5.4), with torsional degrees of freedom defined and all acyclic dihedral angles allowed to rotate freely.

The 3D coordinates of *Ee*AChE (PDB ID: 1C2B) were retrieved from the Protein Data Bank. Protein preparation involved removal of water molecules, heteroatoms, co-crystallized solvent, and the bound ligand, followed by assignment of bond types, bond orders, hybridization states, and charges using the Discovery Studio 2023 protein modeling tool. The CHARMm force field was applied for receptor–ligand interaction modeling. Hydrogens and Gasteiger partial charges were added to both protein and ligands via ADT. Eight residues lining the AChE binding site (Trp286, Tyr124, Tyr337, Tyr72, Asp74, Thr75, Trp86, and Tyr341) were set as flexible using the AutoTors module. The docking box was centered and visualized in ADT, with dimensions of 60 × 60 × 72 Å^3^ and 1 Å spacing (centered at x = 21.5911, y = 87.752, z = 23.591).

For eqBuChE, a homology model was obtained from the SWISS-MODEL Repository [36], using human BuChE (hBuChE) (PDB ID: 2PM8) as a template, which shares 89% sequence identity. Protein preparation and docking followed the same protocol as for *Ee*AChE. The docking box was set to 75 Å^3^ with a grid spacing of 1 Å (centered at x = 29.885, y = −54.992, z = 58.141).

Docking calculations were carried out using AutoDock Vina [35] in blind docking mode, with default parameters except for num_modes, which was set to 40. The conformation with the lowest binding energy was considered the most stable and was subsequently analyzed and visualized in Discovery Studio.

## 3. Materials and Methods

All reagents were obtained from (Merck, Kenilworth, NJ, USA). Melting points were measured using a Kofler apparatus (Wagner Munz, München, Germany). The progress of the reactions was monitored by thin-layer chromatography (TLC) on aluminium plates coated with silica gel 60 F254 (Merck, Kenilworth, NJ, USA). ^1^H and ^13^C NMR spectra were recorded on a Bruker spectrometer operating at 400 MHz and 100 MHz, respectively, with DMSO-d^6^ as the solvent. Chemical shifts (δ) are reported in parts per million (ppm) relative to tetramethylsilane (TMS) as the internal standard. Signal multiplicities are designated as follows: s (singlet), d (doublet), t (triplet), q (quartet), and m (multiplet); coupling constants (J) are expressed in hertz (Hz). High-resolution mass spectra (HRMS) were acquired on a Bruker MicrOTOF-Q II spectrometer (Bruker Daltonics, Bremen, Germany) equipped with a positive electrospray ionization time-of-flight (ESI-TOF) source at the Université Clermont Auvergne (Clermont-Ferrand, France).

### 3.1. Synthesis of Quinazoline Derivatives ***3a**–**q***

In a round-bottom flask equipped with a magnetic stir bar, a mixture of 2-aminobenzonitrile (1.0 equiv) and the substituted orthoester (1.2 equiv) was stirred and heated under reflux for 30 min. After the reaction finished, the solvent was removed under reduced pressure. Subsequently, the substituted aniline (1.0 equiv), xylene, and a catalytic amount of *p*-TsOH were added, and the resulting mixture was stirred and refluxed for 10 h. Upon completion, the mixture was cooled to room temperature (rt), resulting in the formation of a solid, collected by filtration, washed with diethyl ether, and dried to afford the corresponding quinazoline derivative.

**4-anilinoquinazoline (3a)**. Yield = (25%); mp = 230 °C; ^1^H NMR (400 MHz, CDCl_3_) ẟ (ppm): 8.79 (s, 1H), 7.99–7.94 (m, 2H), 7.85–7.81 (m, 1H), 7.77 (d, *J* = 7.7 Hz, 2H), 7.61–7.57 (m, 1H), 7.47–7.43 (m, 2H), 7.23–7.20 (m, 1H). ^13^C NMR (100 MHz, CDCl_3_) ẟ (ppm): 157.7, 154.7, 149.4, 138.0, 133.1, 129.1, 128.5, 126.8, 124.9, 122.1, 120.6, 115.1. IR (KBr) cm^−1^: ν = 3267 (-NH), 1625 (C=N). HRMS (ESI, [M + H]^+^) Calcd for C_14_H_12_N_3_: 222.1025; Found: 222.1026.

**2-Methyl-4-anilinoquinazoline (3b).** Yield = (20%); mp = 235 °C, ^1^H NMR (400 MHz, CDCl_3_) ẟ (ppm): 7.91 (d, *J* = 8.2 Hz, 1H), 7.87–7.83 (m, 3H), 7.77–7.73 (m, 1H), 7.49–7.46 (m, 1H), 7.43–7.40 (m, 2H), 7.18–7.15 (m,1H), 2.72 (s, 3H). ^13^C NMR (100 MHz, CDCl_3_) ẟ (ppm): 164.0, 157.3, 150.4, 138.6, 132.9, 129.0, 128.0, 125.7, 124.1, 121.2, 120.4, 113.2, 26.5. IR (KBr) cm^−1^: ν = 3266 (-NH), 1620 (C=N). HRMS (ESI, [M + H]^+^) Calcd for C_15_H_13_N_3_: 236.10182; Found: 236.1182.

**2-Ethyl-4-anilinoquinazoline (3c).** Yield = (31%); mp = 198 °C, ^1^H NMR (400 MHz, DMSO-*d6*) ẟ (ppm): 9.67 (s, 1H), 8.54 (d, *J* = 7.9 Hz, 1H), 7.97 (d, *J* = 7.6 Hz, 2H), 7.83–7.79 (m, 1H), 7.73 (d, *J* = 8.3 Hz, 1H), 7.58–7.54 (m, 1H), 7.43–7.38 (m, 2H), 7.13–7.09 (m, 1H), 2.80 (q, *J* = 7.6 Hz, 2H), 1.32 (t, *J* = 7.6 Hz, 3H). ^13^C NMR (100 MHz, DMSO-*d6*) ẟ (ppm): 167.2, 158.1, 150.7, 140.0, 133.3, 128.8, 127.9, 125.7, 123.7, 123.3, 122.2, 114.0, 32.8, 12.9. HRMS (ESI, [M + H] ^+^) Calcd for C_16_H_16_N_3_: 250.1338; Found: 250.1338.

**4-(3,5-Dimethoxyphenyl)aminoquinazoline (3d).** Yield = (67%); mp = 160 °C, ^1^H NMR (400 MHz, DMSO-*d6*) ẟ (ppm): 9.67 (s, 1H), 8.65 (s, 1H), 8.57 (d, *J* = 7.8 Hz), 7.89–7.85 (m, 1H), 7.80 (d, *J* = 8.4 Hz, 1H), 7.67–7.63 (m, 1H), 7.25 (d, *J* = 2.2 Hz, 2H), 6.31 (t, *J* = 2.2 Hz, 1H), 3.78 (s, 6H).^13^C NMR (100 MHz, DMSO-*d6*) ẟ (ppm): 160.8, 158.1, 154.8, 150.1, 141.4, 133.5, 128.3, 126.8, 123.4, 115.7, 100.9, 96.0, 55.7. HRMS (ESI, [M + H]^+^) Calcd for C_16_H_16_N_3_O_2_: 282.1237; Found: 282,1232.

**2-methyl-4-(3,5-Dimethoxyphenyl)aminoquinazoline (3e).** Yield = (25%); mp = 109 °C, ^1^H NMR (400 MHz, DMSO-*d6*) ẟ (ppm): 9.56 (s, 1H), 8.53 (d, *J* = 8.0 Hz, 1H), 7.89–7.79 (m, 1H), 7.71 (d, *J* = 7.6 Hz, 1H), 7.58–7.54 (m, 1H), 7.36 (d, *J* = 2.2 Hz, 2H), 6.27 (t, *J* = 2.2 Hz, 1H), 3.78 (s, 6H), 2.55 (s, 3H). ^13^C NMR (100 MHz, DMSO-*d6*) ẟ (ppm): 163.2, 160.7, 158.0, 150.7, 141.7, 133.3, 127.8, 125.8, 123.2, 113.8, 100.2, 95.9, 55.6, 26.6. HRMS (ESI, [M + H]^+^) Calcd for C_17_H_18_N_3_O_2_: 296.1392; Found: 296,1393.

**2-ethyl-4-(3,5-Dimethoxyphenyl)aminoquinazoline (3f).** Yield = (26%); mp = 138 °C, ^1^H NMR (400 MHz, DMSO-*d6*) ẟ (ppm): 9.56 (s, 1H), 8.54 (d, *J* = 8.3 Hz, 1H), 7.84–7.80 (m, 1H), 7.73 (d, *J* = 8.3 Hz, 1H), 7.59–7.55 (m, 1H), 7.38 (d, *J* = 2.2 Hz, 2H), 6.27 (t, *J* = 2.2 Hz, 1H), 3.78 (s, 6H), 2.83 (q, *J* = 7.6 Hz, 2H), 1.35 (t, *J* = 7.6 Hz, 3H). ^13^C NMR (100 MHz, DMSO-*d6*) ẟ (ppm): 167.1, 160.7, 158.1, 150.7, 141.8, 133.3, 127.9, 125.8, 123.2, 114.0, 100.0, 95.9, 55.6, 32.8, 12.9. HRMS (ESI, [M + H]^+^) Calcd for C_18_H_20_N_3_O_2_: 310.1550; Found: 310,1546.

**4-(3,4-Dimethoxyphenyl)aminoquinazoline (3g).** Yield = (55%); mp = 190 °C, ^1^H NMR (400 MHz, DMSO-*d6*) ẟ (ppm): 9.68 (s, 1H), 8.56 (s, 1H,), 8.53 (d, *J* = 8.4 Hz, 1H), 7.87–7.82 (m, 1H), 7.77 (d, *J* = 8.3 Hz, 1H), 7.64–7.60 (m, 1H), 7.47 (d, *J* = 2.4 Hz, 1H), 7.43 (dd, *J* = 8.6 Hz, 2.4 Hz), 6.99 (d, *J* = 8.6 Hz, 1H), 3.79 (s, 3H), 3.78 (s, 3H). ^13^C NMR (100 MHz, DMSO-*d6*) ẟ (ppm): 158.3, 155.1, 150.0, 148.9, 146.0, 133.3, 132.9, 128.2, 126.5, 123.3, 115.6, 115.4, 112.2, 108.3, 56.2, 56.0. HRMS (ESI, [M + H]^+^) Calcd for C_16_H_16_N_3_O_2_: 282.1234; Found: 282,1237.

**2-ethyl-4-(3,4-Dimethoxyphenyl)aminoquinazoline (3h).** Yield = (35%); mp = 178 °C, ^1^H NMR (400 MHz, DMSO-*d6*) ẟ (ppm): 9.57 (s, 1H), 8.50 (d, *J* = 8.1 Hz, 1H), 7.69–7.81 (m, 3H), 7.56–7.52 (m, 1H), 7.46 (d, *J* = 8.5 Hz, 1H), 6.98 (d, *J* = 8.5 Hz, 1H), 3.81 (s, 3H), 3.77 (s, 3H), 2.78 (q, *J* = 7.5 Hz, 2H), 1.32 (t, *J* = 7.5 Hz, 3H). ^13^C NMR (100 MHz, DMSO-*d6*) ẟ (ppm): 167.3, 158.0, 150.6, 148.7, 145.4, 133.4, 133.1, 127.8, 125.6, 123.1, 114.1, 114.0, 112.2, 107.5, 56.2, 55.8, 32.8, 12.9. HRMS (ESI, [M + H]^+^) Calcd for C_18_H_20_N_3_O_2_: 310.1550; Found: 310,1548.

**4-(*p*-Tolyl)aminoquinazoline (3i).** Yield = (30%); mp = 198 °C, ^1^H NMR (400 MHz, CDCl3) ẟ (ppm): 8.74 (s, 1H), 7.92–7.89 (m, 2H), 7.79–7.75 (m, 1H), 7.62 (s, 1H), 7.57 (d, *J* = 8.2 Hz, 2H), 7.54–7.50 (m, 1H), 7.20 (d, *J* = 8.2 Hz, 2H), 2.35 (s, 3H). ^13^C NMR (100 MHz, CDCl_3_) ẟ (ppm): 157.9, 155.1, 149.8, 135.4, 134.7, 132.9, 129.7, 128.8, 126.5, 122.5, 120.5, 115.1, 21.0. HRMS (ESI, [M + H]^+^) Calcd for C_15_H_14_N_3_: 236.1182; Found: 236.1181.

**2-Methyl-4-(*p*-tolyl)aminoquinazoline** (**3j**). Yield = (35%); mp = 210 °C, ^1^H NMR (400 MHz, CDCl_3_) ẟ (ppm): 7.89–7.84 (m, 2H), 7.77–7.73 (m, 1H), 7.70 (d, *J* = 8.2 Hz, 2H), 7.57 (s, 1H), 7.50–7.46 (m, 1H), 7.22 (d, *J* = 8.2 Hz, 2H), 2.71 (s, 3H), 2.38 (s, 3H). ^13^C NMR (100 MHz, CDCl_3_) ẟ (ppm): 164.1, 157.3, 150.3, 135.9, 133.9, 132.8, 129.5, 128.0, 125.6, 121.3, 120.3, 113.1, 26.6, 21.0. HRMS (ESI, [M + H]^+^) Calcd for C_16_H_16_N_3_: 250.1338; Found: 250.1337.

**2-Ethyl-4-(*p*-tolyl)aminoquinazoline (3k**). Yield = (39%); mp: 212 °C, ^1^H NMR (400 MHz, CDCl_3_) ẟ (ppm): 8.05 (d, *J* = 6.2 Hz, 1H), 7.90 (d, *J* = 6.7 Hz), 7.77–7.72 (m, 3H), 7.51–7.48 (m, 1H), 7.22 (d, *J* = 6.7 Hz, 2H), 2.97 (q, *J* = 7.6 Hz, 2H, CH_2_), 2.38 (s, 3H), 1.42 (t, *J* = 7.6 Hz, 3H). ^13^C NMR (100 MHz, CDCl_3_) ẟ (ppm): 168.27, 157.33, 150.6, 136.1, 133.6, 132. 7, 129.5, 128.4, 125.6, 121.1, 120.2, 113.4, 33.2, 20.9, 12.8. HRMS (ESI, [M + H]^+^) Calcd for C_17_H_18_N_3_: 264.1495; Found: 264.1495.

**4-(2,3-Dimethylphenyl)aminoquinazoline (3l).** Yield = (62%); mp = 184 °C, ^1^H NMR (400 MHz, DMSO-*d6*) ẟ (ppm): 9.73 (s, 1H), 8.48 (d, *J* = 8.4 Hz, 1H), 8.39 (s, 1H), 7.86–7.82 (m, 1H), 7.76 (d, *J* = 8.4 Hz), 7.63–7.59 (m, 1H), 7.17–7.12 (m, 3H), 2.31 (s, 3H), 2.07 (s, 3H). ^13^C NMR (100 MHz, DMSO-*d6*) ẟ (ppm): 159.6, 155.4, 150.0, 137.7, 137.5, 134.3, 133.3, 128.4, 128.1, 126.5, 126.0, 126.0, 123.5, 115.2, 20.7, 14.8. HRMS (ESI, [M + H]^+^) Calcd for C_16_H_16_N_3_: 250.1338; Found: 250.1337.

**2-methyl-4-(2,3-Dimethylphenyl)aminoquinazoline (3m).** Yield = (30%); mp = 217 °C, ^1^H NMR (400 MHz, CDCl3) ẟ (ppm): 7.87–7.82 (m, 2H), 7.79–7.75 (m, 1H), 7.65 (d, *J* = 7.9 Hz, 1H), 7.50–7.46 (m, 1H), 7.33 (s, 1H), 7.22–7.18 (m, 1H), 7.11 (d, *J* = 7.5 Hz, 1H), 2.64 (s, 3H), 2.38 (s, 3H), 2.25 (s, 3H). ^13^C NMR (100 MHz, CDCl3) ẟ (ppm): 164.4, 158.1, 150.7, 137.7, 136.4, 132.8, 130.7, 128.2, 127.5, 125.8, 125.5, 122.8, 120.6, 113.1, 26.7, 20.7, 14.3. HRMS (ESI, [M + H]^+^) Calcd for C_17_H_18_N_3_ [M + H]^+^: 264.1495; Found: 264.1493.

**4-(4-Nitrophenyl)aminoquinazoline (3n).** Yield = (78%); mp > 260 °C, ^1^H NMR (400 MHz, DMSO-*d6*) ẟ (ppm): 10.31 (s, 1H), 8.79 (s, 1H), 8.64 (d, *J* = 6.7 Hz, 1H), 8.32–8.28 (m, 4H), 7.97–7.93 (m, 1H), 7.88 (d, *J* = 6.7 Hz, 1H), 7.75–7.72 (m, 1H).^13^C NMR (100 MHz, DMSO-*d6*) ẟ (ppm): 157.8, 154.4, 150.5, 146.5, 142.4, 134.0, 128.5, 127.3, 125.1, 123.6, 121.3, 115.9. HRMS (ESI, [M + H]^+^) Calcd for C_14_H_11_N_4_O_2_: 267.0876; Found: 267.0874.

**2-Methyl-4-(4-nitrophenyl)aminoquinazoline (3o).** Yield = (25%); mp = 263 °C, ^1^H NMR (400 MHz, DMSO-*d6*) ẟ (ppm): 9.94 (s, 1H), 8.22–8.20 (m, 4H), 7.97–7.99 (m, 2H), 7.05–7.03 (m, 2H), 2.03 (s, 3H). ^13^C NMR (100 MHz, DMSO-*d6*) ẟ (ppm): 157.8, 154.3, 147.2, 142.7, 141.6, 125.3, 122.6, 119.0, 18.9. HRMS (ESI, [M + H]^+^) Calcd for C_15_H_12_N_4_O_2_: 281.1033; Found: 281.1031.

**4-(4-Chlorophenyl)aminoquinazoline (3p).** Yield = (40%); mp = 204 °C, ^1^H NMR (400 MHz, CDCl_3_) ẟ (ppm): 8.79 (s, 1H), 7.96–7.94 (m, 2H), 7.85–7.81 (m, 1H), 7.73 (d, *J* = 8.8 Hz, 2H), 7.69 (s, 1H), 7.60–7.56 (m,1H), 7.39 (d, *J* = 8.8 Hz, 2H). ^13^C NMR (100 MHz, CDCl_3_) ẟ (ppm): 157.4, 154.7, 149.8, 136.7, 133.1, 129.7, 129.1, 129.0, 126.9, 123.1, 120.1, 115.0. HRMS (ESI, [M + H]^+^) Calcd for C_14_H_11_N_3_Cl: 256.0636; Found: 256.0636.

**2-ethyl-4-(4-Chlorophenyl)aminoquinazoline (3q).** Yield = (20%); mp = 164 °C, ^1^H NMR (400 MHz, DMSO-*d6*) ẟ (ppm): 9.77 (s, 1H), 8.52 (d, *J* = 8.2 Hz, 1H), 8.03 (d, *J* = 8.9 Hz, 2H), 7.84–7.80 (m, 1H), 7.74 (d, *J* = 7.7 Hz, 1H), 7.59–7.55 (m, 1H), 7.45 (d, *J* = 8.9 Hz, 2H), 2.81 (q, *J* = 7.6 Hz, 1H), 1.32 (t, *J* = 7.6 Hz, 3H). ^13^C NMR (100 MHz, DMSO-*d6*) ẟ (ppm): 167.1, 158.0, 150.7, 134.0, 133.4, 128.7, 127.9, 127.2, 125.9, 123.6, 123.3, 113.3, 32.8, 12.9. HRMS (ESI, [M + H]^+^) Calcd for C_16_H_15_N_3_Cl: 284.0946; Found: 284.0949.

### 3.2. Biological Evaluation

#### 3.2.1. Inhibition of *Ee*AChE and eqBuChE

The inhibitory activities of the tested compounds against *Ee*AChE and eqBuChE were determined using the spectrophotometric procedure based on Ellman’s method [27]. Reactions were carried out in a total volume of 3 mL of 0.1 M phosphate buffer (pH 8.0) containing 5,5′-dithiobis(2-nitrobenzoic acid) (DTNB; 2625 µL, final concentration 0.35 mM), *Ee*AChE (29 µL, final concentration 0.035 U/mL) or eqBuChE (60 µL, final concentration 0.05 U/mL), the test compound (3 µL, 0.001–1000 nM final concentrations), and bovine serum albumin (BSA; 60 µL, 1% w/v in phosphate buffer, pH 8). After a pre-incubation step, acetylthiocholine iodide (105 µL, final concentration 0.35 mM) or butyrylthiocholine iodide (150 µL, final concentration 0.5 mM) was added, and the mixture was incubated for an additional 15 min. For IC_50_ determination, inhibition curves were obtained by pre-incubating the reaction mixture at room temperature with nine concentrations of each compound for 10 min.

#### 3.2.2. Oxygen Radical Absorbance Capacity Assay

The antioxidant activities of compounds **3a**–**q** were assessed using the ORAC-FL assay with fluorescein as the fluorescent probe. Briefly, fluorescein and the test compound were pre-incubated in a black 96-well microplate (Nunc) at 37 °C for 15 min. Then, 2,2′-azobis(amidinopropane) dihydrochloride was rapidly added using the integrated injector of a Varioskan Flash plate reader (Thermo Scientific). Fluorescence readings were recorded at an excitation wavelength of 485 nm and an emission wavelength of 535 nm at 1 min intervals over 60 min. Each experiment was conducted in triplicate, with a minimum of three independent assays performed per compound.

#### 3.2.3. Self-Mediated Aggregation of Aβ_1–42_

A ThT fluorescence assay evaluated the effect of the test compounds on Aβ_1−42_ fibril formation. Aβ stock solutions were diluted to 50 μM in ultrapure water before use. For each assay, 20 μL of the peptide (final concentration 25 μM) was incubated at 37 °C for 24 h either in the presence or absence of 20 μL of the test compound (final concentration 20 μM). Control samples were prepared by replacing Aβ with 50 mM phosphate buffer (pH 7.4), with or without the inhibitors. After incubation, samples were diluted to 360 μL with 50 mM glycine-NaOH buffer (pH 8.0) containing thioflavin T (5 μM). Fluorescence intensities were recorded after 5 min (excitation at 450 nm, emission at 485 nm). The percentage of aggregation inhibition was calculated using the Equation:

% Inhibition = (1 − IFi/IFc) × 100% (1)

where IFi and IFc represent the fluorescence intensities of Aβ in the presence and absence of inhibitors, respectively, after subtraction of background signals

#### 3.2.4. Molecular Docking of Compounds **3e** and **3h** into *Ee*AChE and eqBuChE

Compounds **3e** and **3h** were constructed in *Discovery Studio 2023* using standard bond lengths and angles. The molecular geometries of **3e** and **3h** were energy-minimized using the adopted-based Newton–Raphson algorithm, employing the CHARMm force field [37] and partial atomic charges. Structures were considered fully optimized when the energy changes between successive iterations were less than 0.001 kcal/mol [38]. Ligands were prepared for docking using AutoDockTools (ADT version 1.5.4), where torsional degrees of freedom were defined, allowing all acyclic dihedral angles to rotate freely. The 3D coordinates of *Ee*AChE (PDB ID: 1C2B) were obtained from the Protein Data Bank (PDB). Protein preparation involved the removal of all water molecules, heteroatoms, co-crystallized solvent, and ligands, followed by assignment of bonds, bond orders, hybridization states, and charges using the Discovery Studio protein modeling tool. The CHARMm force field was applied to model receptor–ligand interactions using the Discovery Studio software package. Hydrogens and Gasteiger partial charges were added to both proteins and ligands via ADT (version 1.5.4). To introduce flexibility into the binding site, eight residues (Trp286, Tyr124, Tyr337, Tyr72, Asp74, Thr75, Trp86, and Tyr341) lining the AChE binding site were allowed to move using the AutoTors module. The docking box was visualized in ADT and was positioned in the middle of the protein, centered at x = 21.591, y = 87.752, z = 23.591, with dimensions of 60 × 60 × 72 Å^3^ and a grid spacing of 1 Å. For eqBuChE, a homology model was retrieved from the SWISS-MODEL Repository [36], generated using the crystal structure of hBuChE (PDB ID: 2PM8) as a template, sharing 89% sequence identity with the equine enzyme. Initial protein preparation and docking calculations followed the same procedure used for *Ee*AChE. The grid box was defined as a 75 Å^3^ cube with 1 Å grid spacing, centered at x = 29.885, y = −54.992, z = 58.141. Docking calculations were performed using AutoDock Vina [35] in blind docking mode, with default parameters except for num_modes, which was set to 40. The conformation with the lowest predicted docking energy was considered the most stable. Docking results were analyzed and visualized in Discovery Studio 2023.

## 4. Conclusions

In this study, a new series of quinazoline derivatives **3a**–**q** was synthesized and biologically evaluated as potential multifunctional agents for AD therapy. Several compounds exhibited noteworthy biological activities, including antioxidant capacity, ChE inhibition, and suppression of Aβ_1–42_ self-aggregation. Among them, 2-ethyl-4-(3,4-Dimethoxyphenyl)aminoquinazoline (**3h**) emerged as the most promising hit, displaying the highest antioxidant activity (ORAC = 5.73 TE), the strongest inhibition of Aβ_1−42_ aggregation (36.68%), and significant AChE inhibition (IC_50_ = 6.61 µM). These results, further supported by molecular modeling insights that elucidate the binding interactions underlying the observed activities, highlight the potential of the quinazoline scaffold as a versatile platform for the development of MTDLs capable of simultaneously addressing oxidative stress, cholinergic dysfunction, and Aβ aggregation, which are key features of the multifactorial pathology of AD. Future work will focus on structural optimization aimed at enhancing ChE inhibition while preserving antioxidant and anti-Aβ properties, combined with in silico approaches, to identify potent neuroprotective candidates for further preclinical evaluation and therapeutic development.

## Figures and Tables

**Table 1:** ChE inhibition (IC_50_ for *Ee*AChE and eqBuChE), and antioxidant capacity (ORAC values) of quinazolines **3a**–**q**.

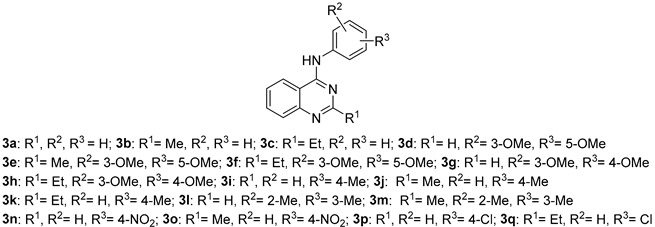						
Quinazolines/ Standards	R^1^	R^2^	R^3^	IC_50_ *Ee*AChE (μM) ^a^	IC_50_ eqBuChE (μM) ^a^	ORAC ^b^
**3a**	H	H	H	-^c^	-^c^	0.20 ± 0.04
**3b**	CH_3_	H	H	-^c^	-^c^	0.51 ± 0.02
**3c**	C_2_H_5_	H	H	-^c^	5.34 ± 0.15	0.15 ± 0.03
**3d**	H	3-OCH_3_	5-OCH_3_	-^c^	-^c^	2.85 ± 0.62
**3e**	CH_3_	3-OCH_3_	5-OCH_3_	6.04 ± 1.05	9.43 ± 1.13	1.81 ± 0.19
**3f**	C_2_H_5_	3-OCH_3_	5-OCH_3_	-^c^	8.50 ± 1.16	1.29 ± 0.08
**3g**	H	3-OCH_3_	4-OCH_3_	-^c^	-^c^	5.46 ± 0.11
**3h**	C_2_H_5_	3-OCH_3_	4-OCH_3_	6.61 ± 0.11	-^c^	5.73 ± 0.39
**3i**	H	H	4-CH_3_	-^c^	-^c^	0.43 ± 0.09
**3j**	CH_3_	H	4-CH_3_	-^c^	-^c^	0.75 ± 0.08
**3k**	C_2_H_5_	H	4-CH_3_	-^c^	-^c^	0.58 ± 0.04
**3l**	H	2-CH_3_	3-CH_3_	-^c^	-^c^	0.32 ± 0.05
**3m**	CH_3_	2-CH_3_	3-CH_3_	-^c^	-^c^	0.45 ± 0.03
**3n**	H	H	4-NO_2_	-^c^	-^c^	0.29 ± 0.02
**3o**	CH_3_	H	4-NO_2_	-^c^	-^c^	0.36 ± 0.01
**3p**	H	H	4-Cl	-^c^	-^c^	0.40 ± 0.07
**3q**	C_2_H_5_	H	4-Cl	-^c^	8.64 ± 0.30	0.25 ± 0.05
**Tacrine**				0.03 ± 0.01	5.1 ± 0.1 (nM)	-^d^
**Donepezil**				0.02 ± 0.00	0.8 ± 0.2	-^d^
**Melatonin**				-^d^	-^d^	2.45 ± 0.09

^a^ Each IC_50_ value is expressed as the mean ± SEM from quadruplicates of at least three independent experiments; The values are reported as mean ± SD of at least three independent measurements. ^b^ Data are expressed as Trolox equivalents and represent the mean (n = 3) ± SEM.^c^ % inhibition at 10 μM was below 50%. ^d^ Not determined.

## Data Availability

All data are in supporting information.

## Supplementary Materials

The following supporting information can be downloaded at: https://www.mdpi.com/article/10.3390/molecules30193930/s1, Figures S1–S17: 1H NMR and 13C NMR spectra for Compounds **3a**–**q**; Figures S18 and S19: HSQC and HMBC spectra for Compound **3a;** Figures S20 and S21: HSQC and HMBC spectra for Compound **3d**; Figures S22–S38: HRMS spectra for Compounds **3a**–**q**.
