# Supplementary material for: Multicomponent Synthesis of Multi-Target Quinazolines Modulating Cholinesterase, Oxidative Stress, and Amyloid Aggregation Activities for the Therapy of Alzheimer’s Disease
Source: Molecules. 2025 Sep 30;30(19):3930. doi: 10.3390/molecules30193930 (PMC12526203; doi:10.3390/molecules30193930)
Supplement: Supplementary file 1 [file molecules-30-03930-s001.zip › molecules-3878448-supplementary.pdf]

Supporting Information

Multicomponent Synthesis of Multi-Target Quinazolines Modulating Cholinesterase, Oxidative Stress, and Amyloid Aggregation Activities for the Therapy of Alzheimer's Disease

Saída Chakhari [1,2], José Marco-Contelles [3], Isabel Iriepa [4], Maria do Carmo Carreiras[5], Fakher Chabchoub [1,*] Lhassane Ismaili [2,*] and Bernard Refouvelet [2]

1 Laboratory of Applied Chemistry: Heterocycles, Lipids and Polymers, Faculty of Sciences of Sfax, University of Sfax. B. P 802. 3000 Sfax-Tunisia
2 Université Marie et Louis Pasteur, INSERM UMR1322 LINC, Besançon, F-25000, France
3 Laboratory of Medicinal Chemistry (IQOG, CSIC) C/ Juan de la Cierva 3, 28006-Madrid, Spain
4 Universidad de Alcalá, Departamento de Química Orgánica y Química Inorgánica, Instituto de Investigación Química "Andrés M. del Río" (IQAR), 28805-Alcalá de Henares, Madrid, Spain
5 Research Institute for Medicines (iMed.ULisboa), Faculty of Pharmacy, Universidade de Lisboa, Av. Professor Gama Pinto, 1649-003 Lisboa, Portugal
* Correspondence: LI; lhassane.ismaili@univ-fcomte.fr, FC: fakher.chabchoub@yahoo.fr ;

^{1}H NMR and ^{13}C NMR spectra for Compounds **3a-q**…………………………….....S2-S18

HSQC and HMBC spectra for Compound **3a**…………………………………..……S19

HSQC and HMBC spectra for Compound **3d**…………………………………..……S20

HRMS spectra for Compounds **3a-q**……………………………………………S21-S29

Figure S1. ^{1}H NMR and ^{13}C NMR of 4-anilinoquinazoline 3a

^{1}H NMR of compound **3a**

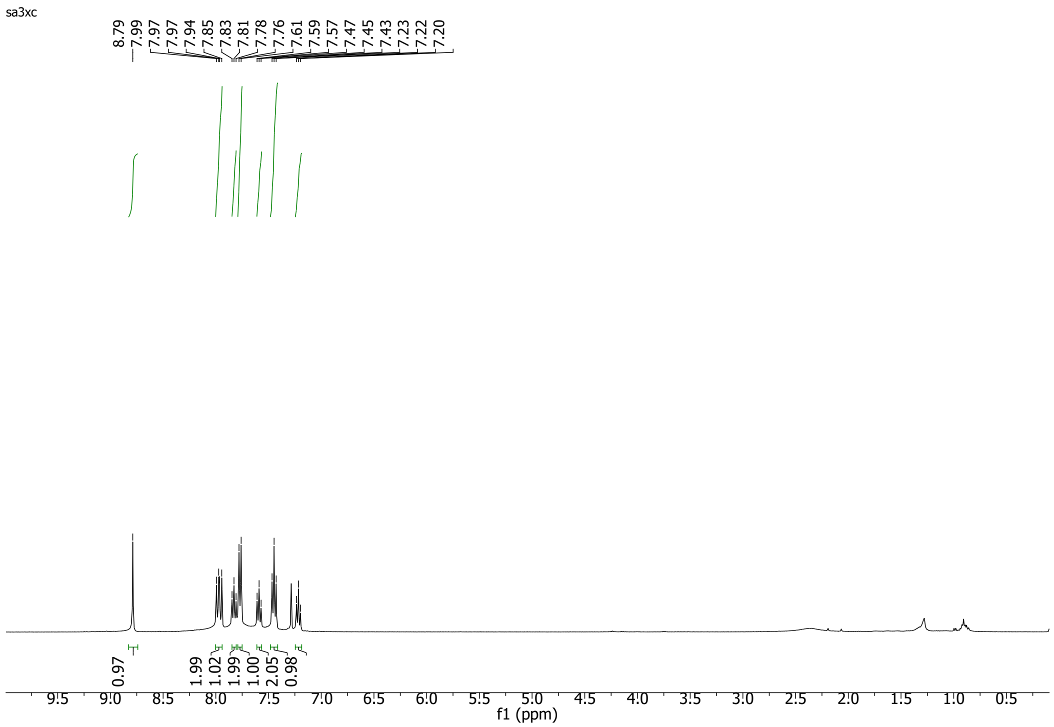

^{13}C NMR of compound **3a**

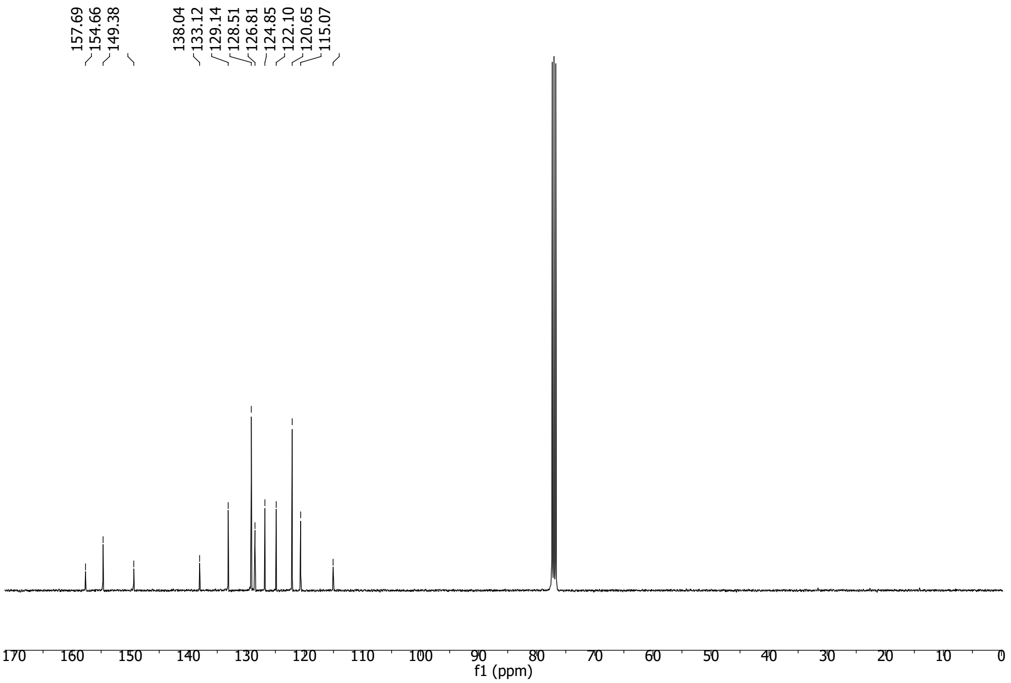

Figure S2. ^{1}H NMR and ^{13}C NMR of 2-Methyl-4-anilinoquinazoline 3b

^{1}H NMR of compound **3b**

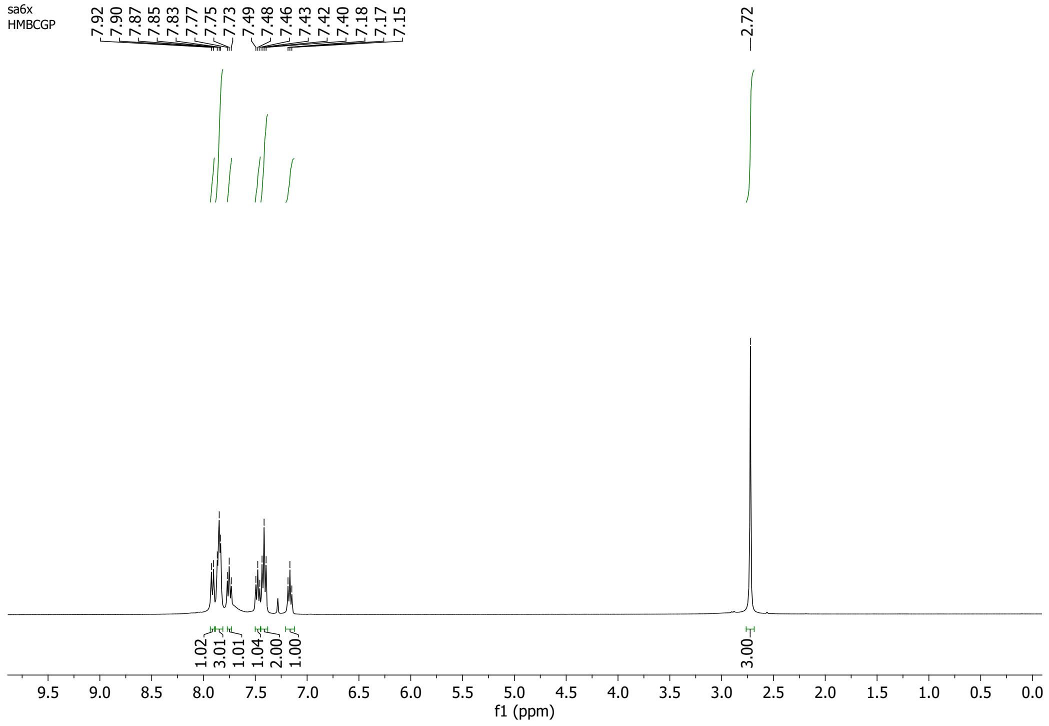

^{13}C NMR of compound **3b**

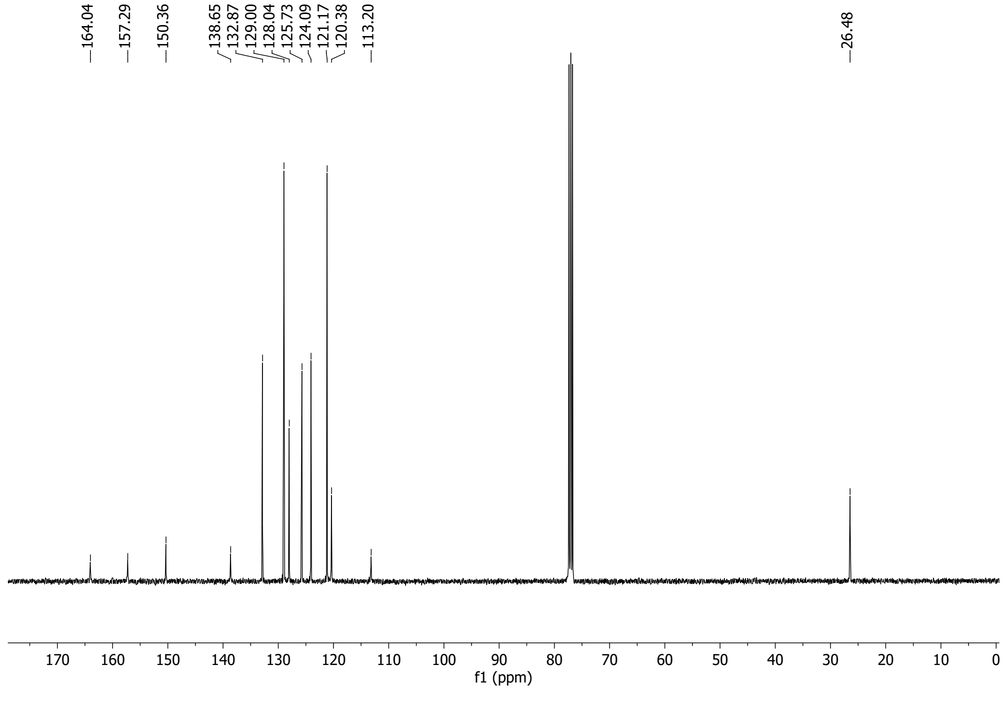

Figure S3. ^{1}H NMR and ^{13}C NMR of 2-Ethyl-4-anilinoquinazoline 3c

^{1}H NMR of compound **3c**

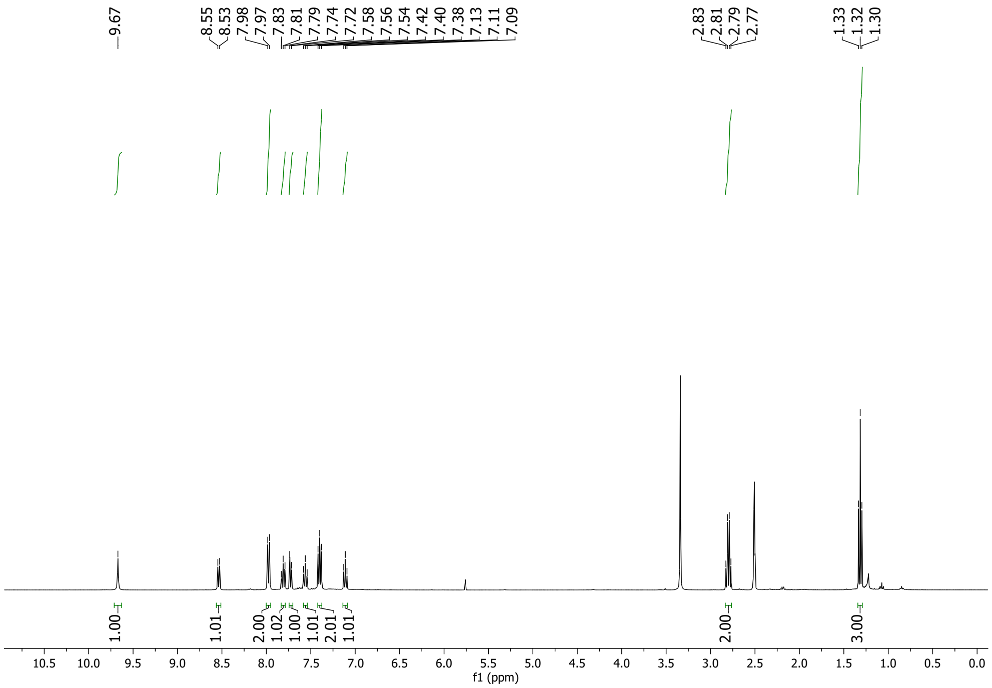

^{13}C NMR of compound **3c**

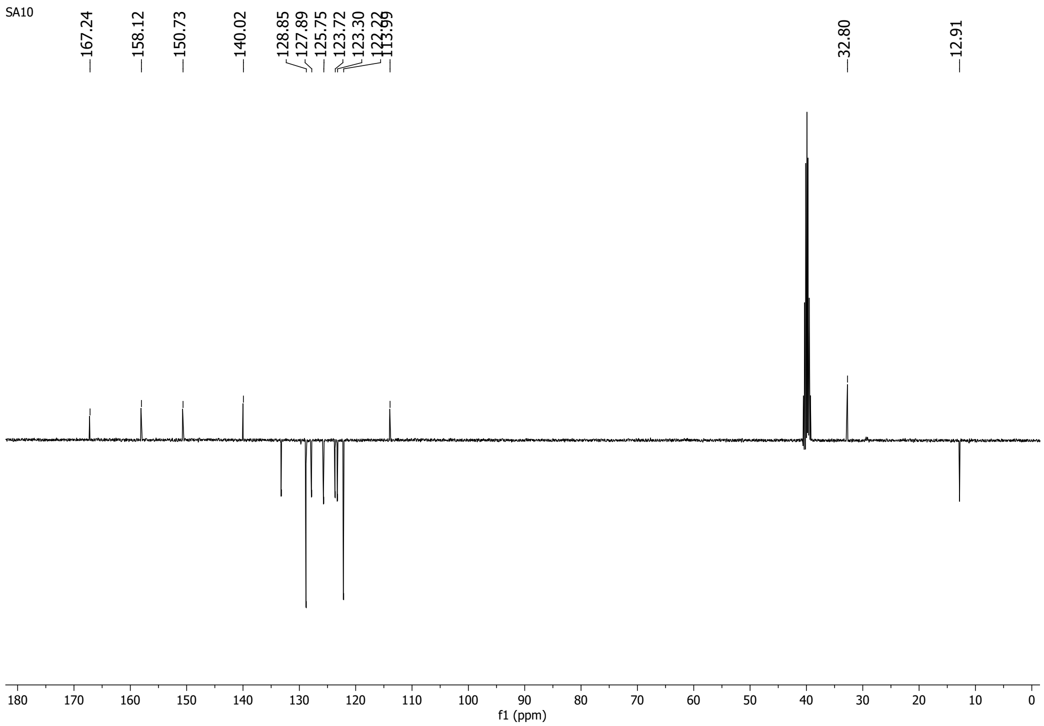

Figure S4. ^{1}H NMR and ^{13}C NMR of 4-(3,5-Dimethoxyphenyl)aminoquinazoline 3d

^{1}H NMR of compound **3d**

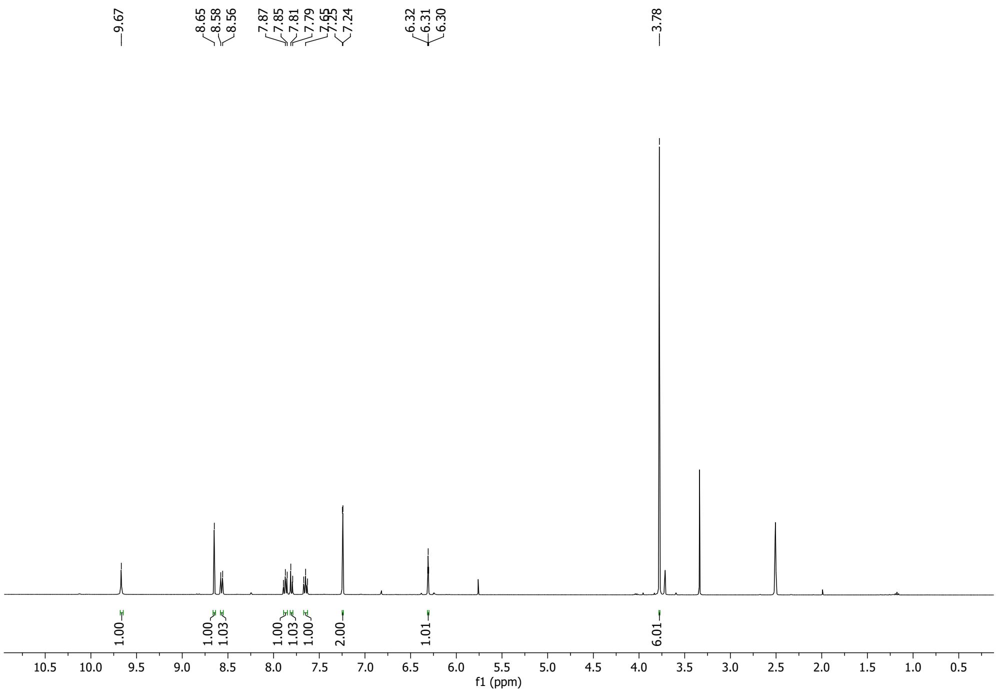

^{13}C NMR of compound **3d**

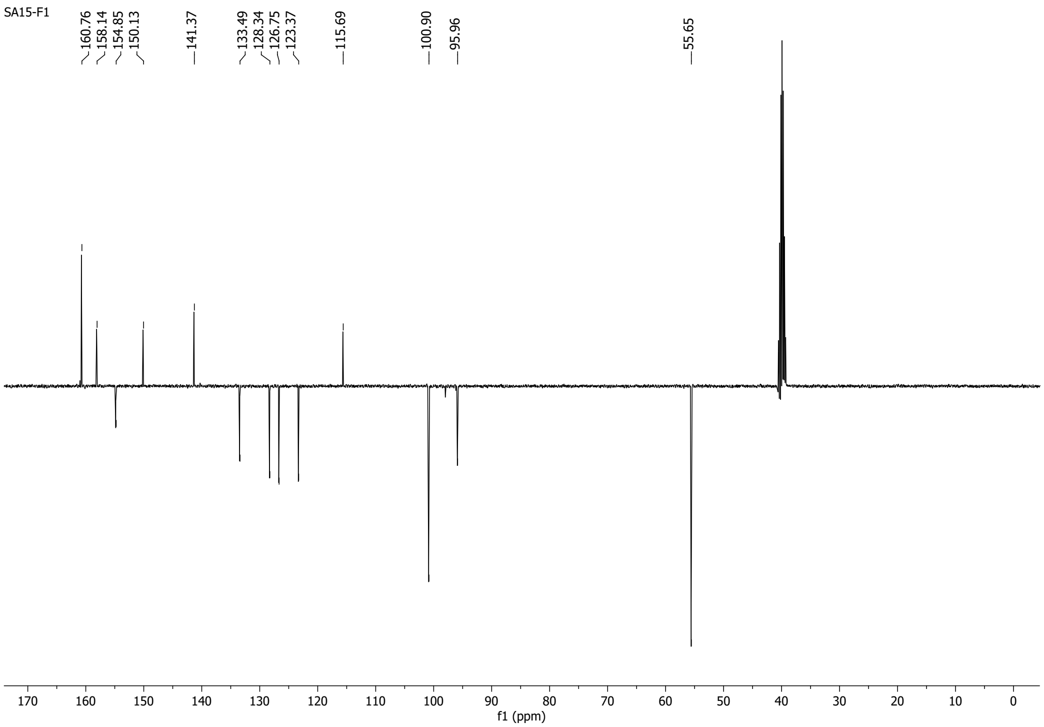

Figure S5. ^{1}H NMR and ^{13}C NMR of 2-methyl-4-(3,5-Dimethoxyphenyl)aminoquinazoline 3e

^{1}H NMR of compound **3e**

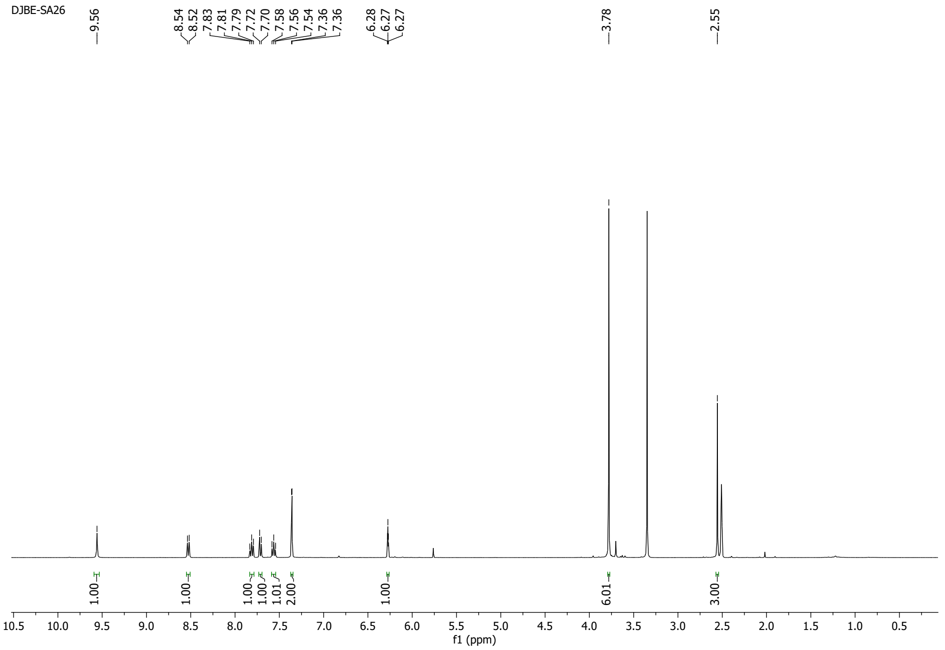

^{13}C NMR of compound **3e**

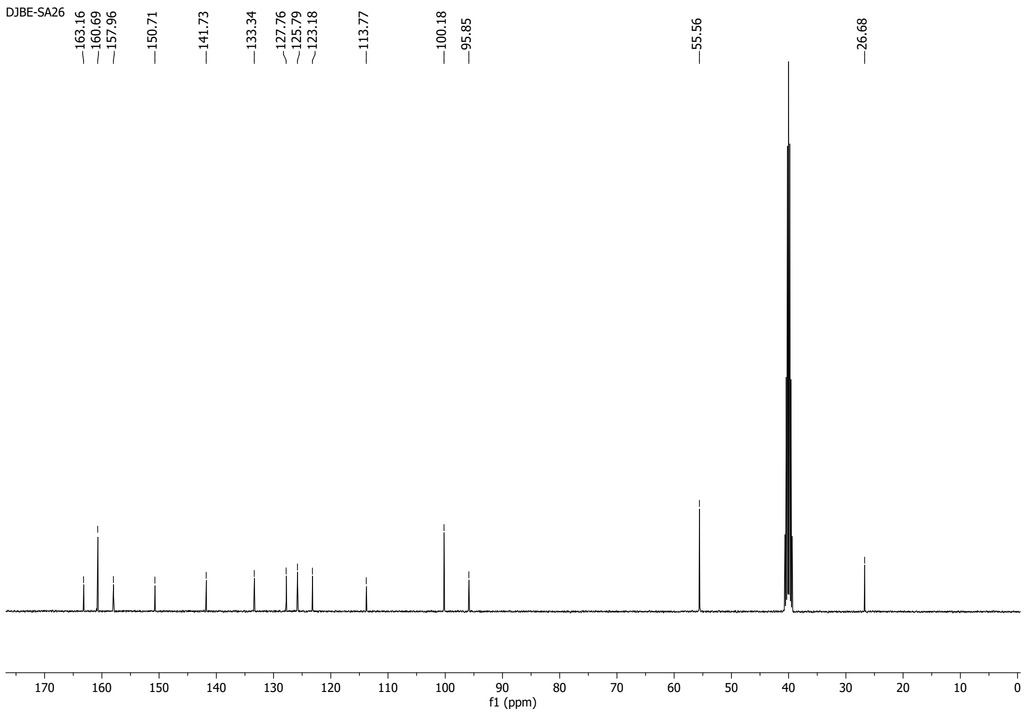

Figure S6. ^{1}H NMR and ^{13}C NMR of 2-ethyl-4-(3,5-Dimethoxyphenyl)aminoquinazoline 3f

^{1}H NMR of compound **3f**

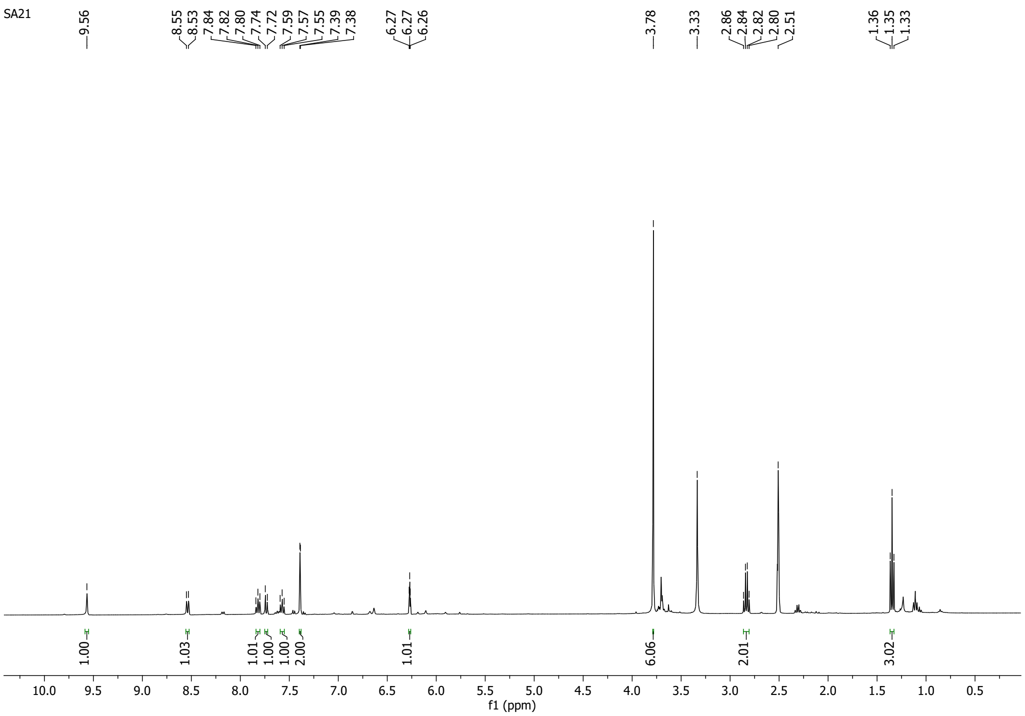

^{13}C NMR of compound **3f**

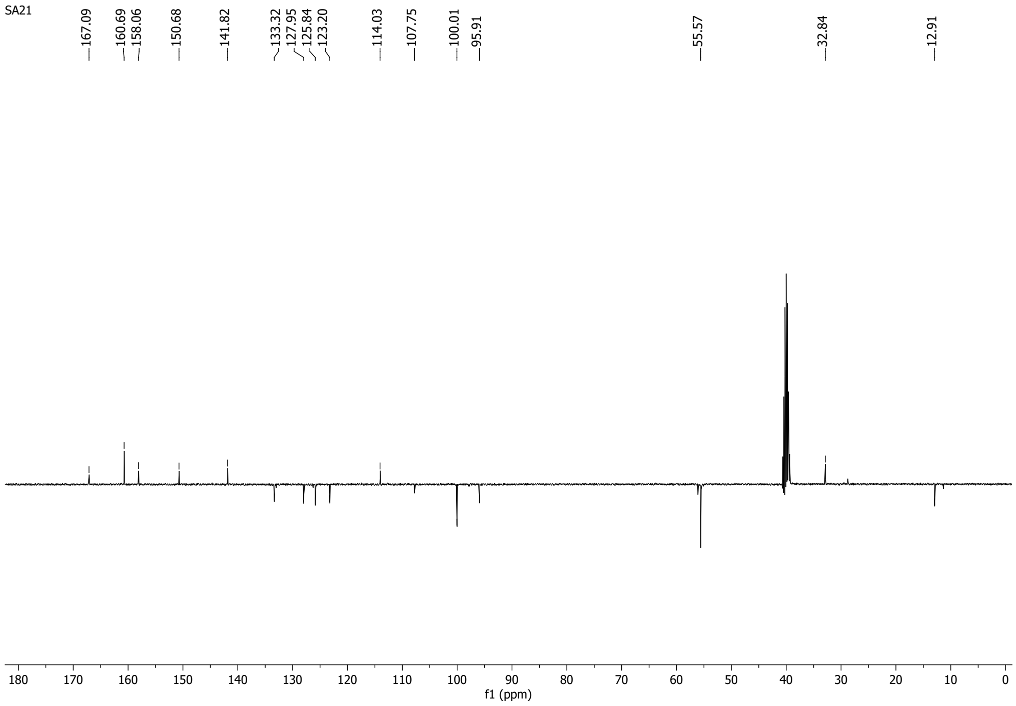

Figure S7. ^{1}H NMR and ^{13}C NMR of 4-(3,4-Dimethoxyphenyl)aminoquinazoline 3g

^{1}H NMR of compound **3g**

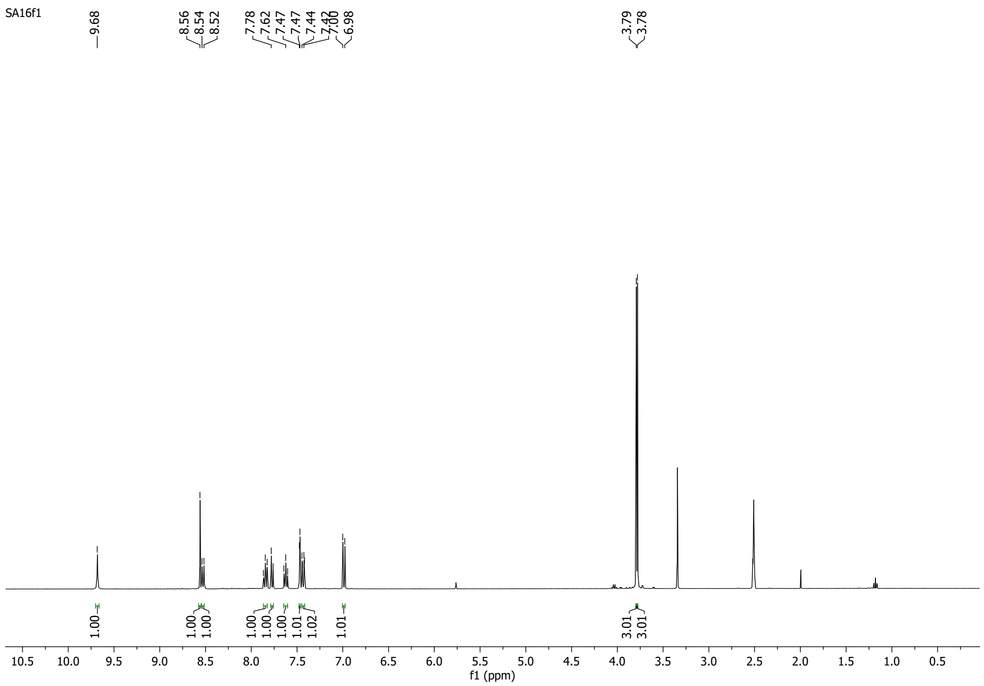

^{13}C NMR of compound **3g**

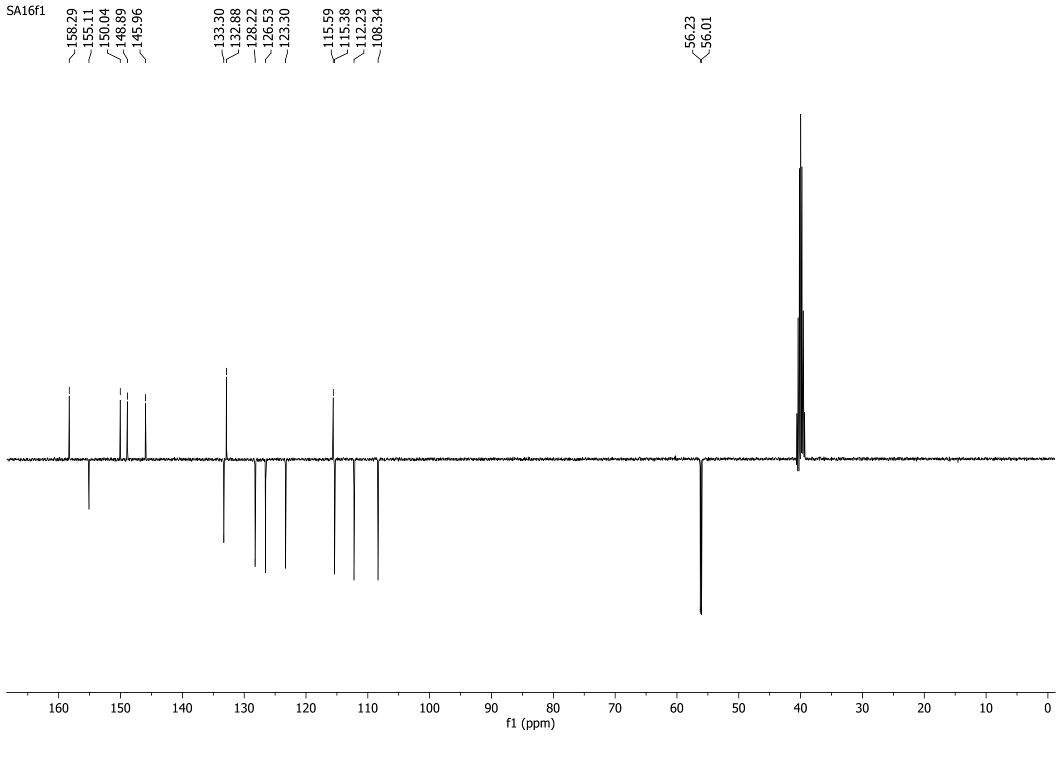

Figure S8. ^{1}H NMR and ^{13}C NMR of 2-ethyl-4-(3,4-Dimethoxyphenyl)aminoquinazoline 3h

^{1}H NMR of compound **3h**

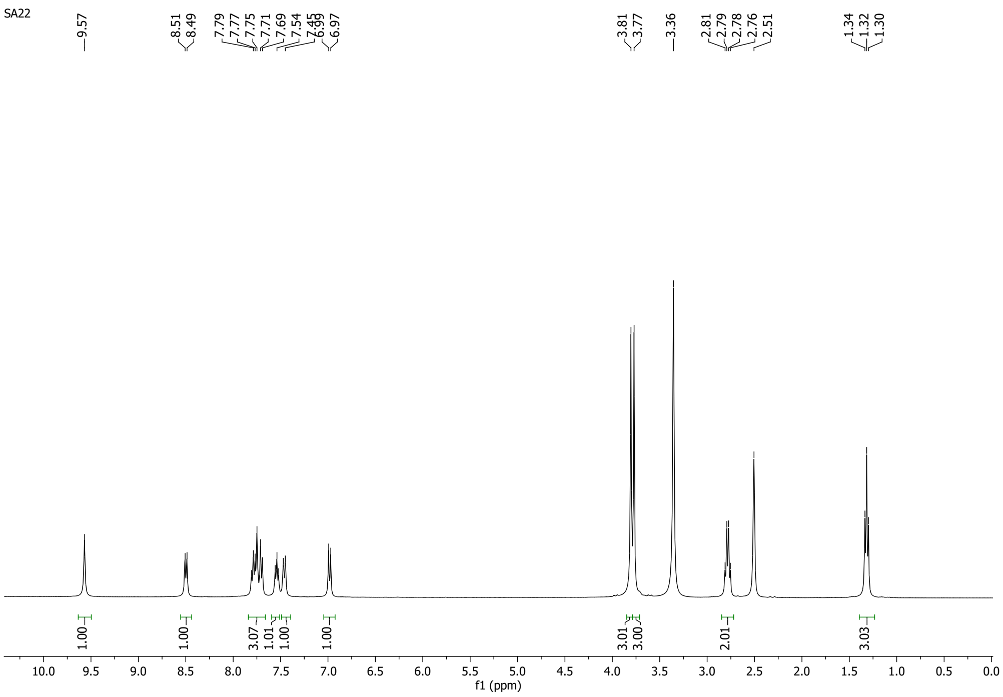

^{13}C NMR of compound **3h**

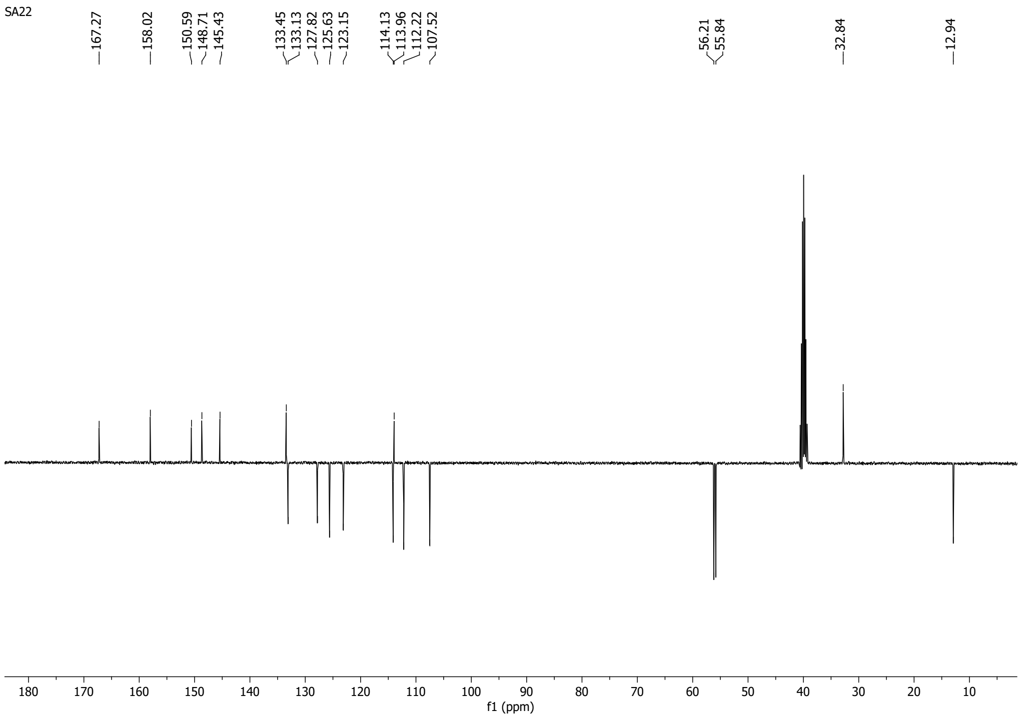

Figure S9. ^{1}H NMR and ^{13}C NMR of 4-(*p*-Tolyl)aminoquinazoline 3i

^{1}H NMR of compound **3i**

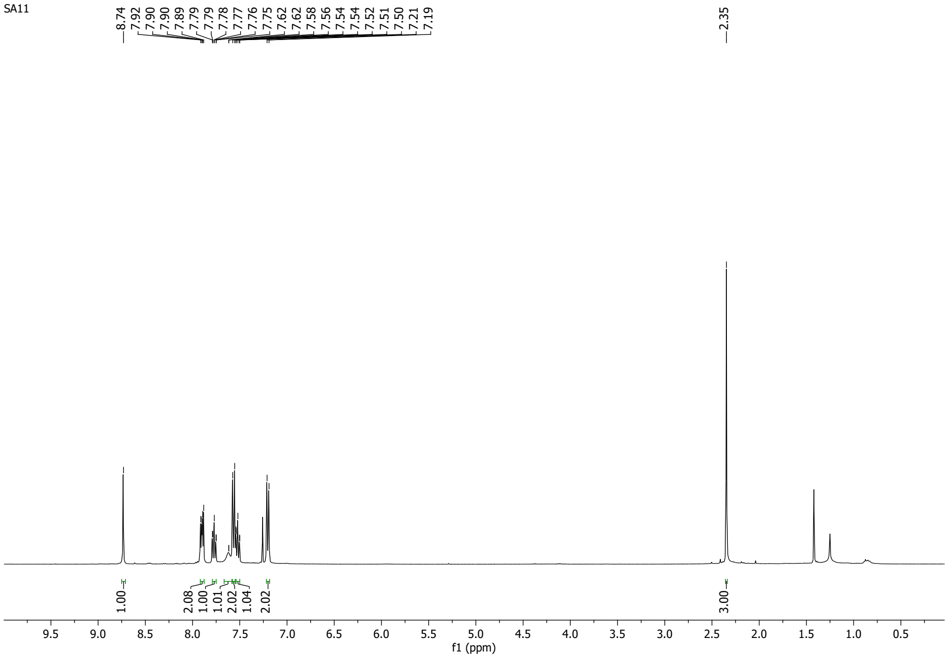

^{13}C NMR of compound **3i**

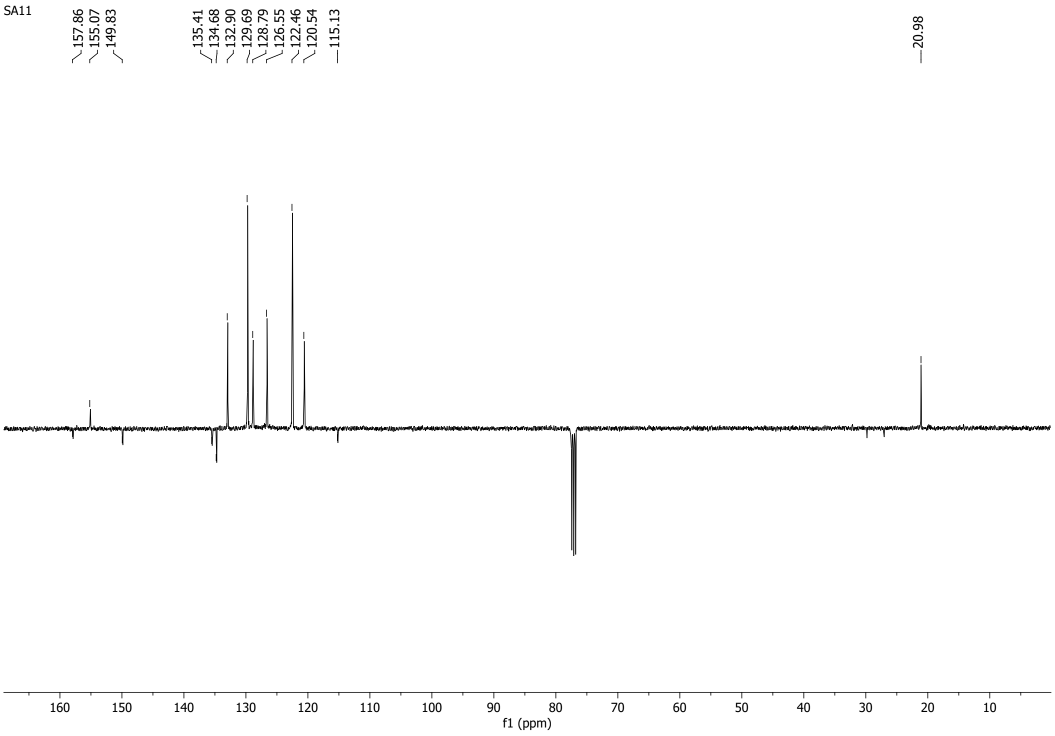

Figure S10. ^{1}H NMR and ^{13}C NMR of 2-Methyl-4-(*p*-tolyl)aminoquinazoline 3j

^{1}H NMR of compound **3j**

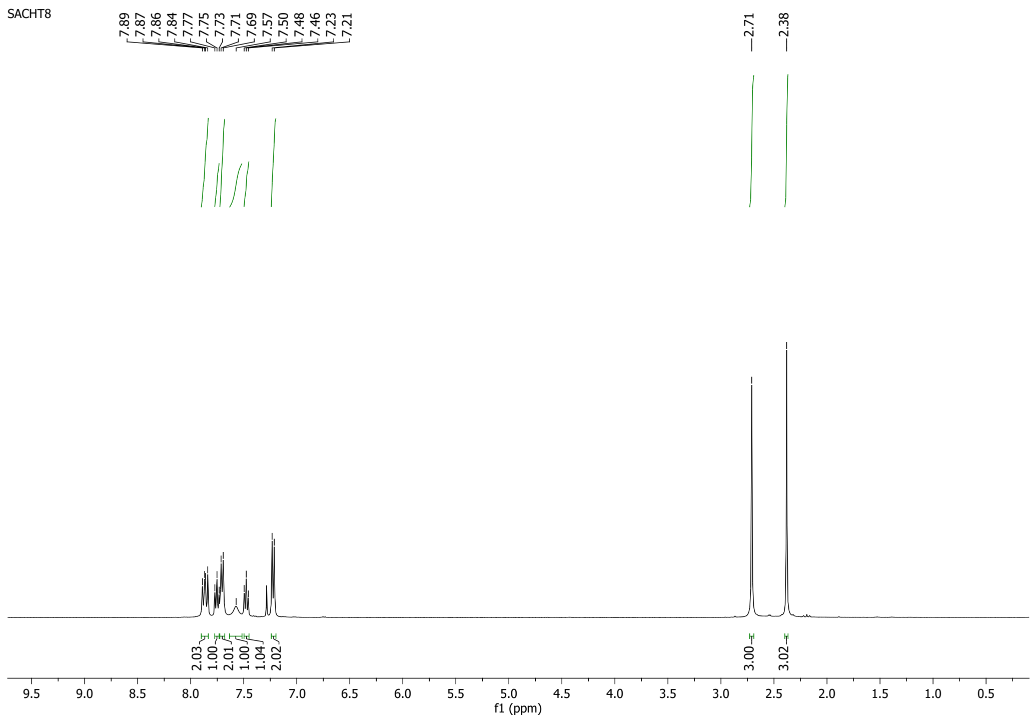

^{13}C NMR of compound **3j**

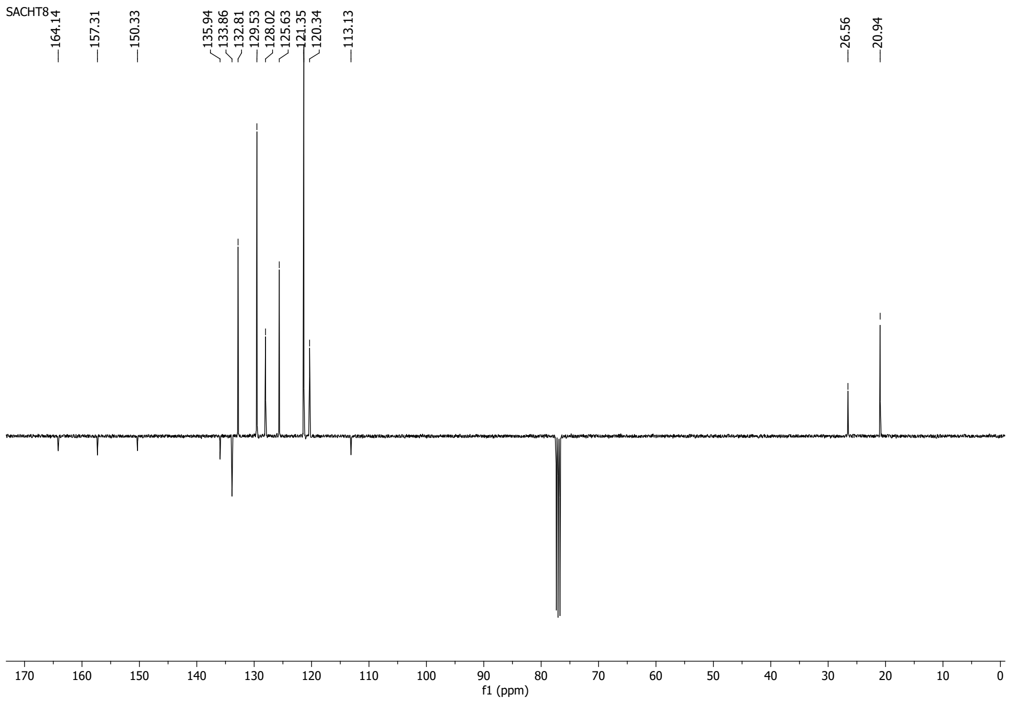

Figure S11. ^{1}H NMR and ^{13}C NMR of 2-Ethyl-4-(*p*-tolyl)aminoquinazoline 3k

^{1}H NMR of compound **3k**

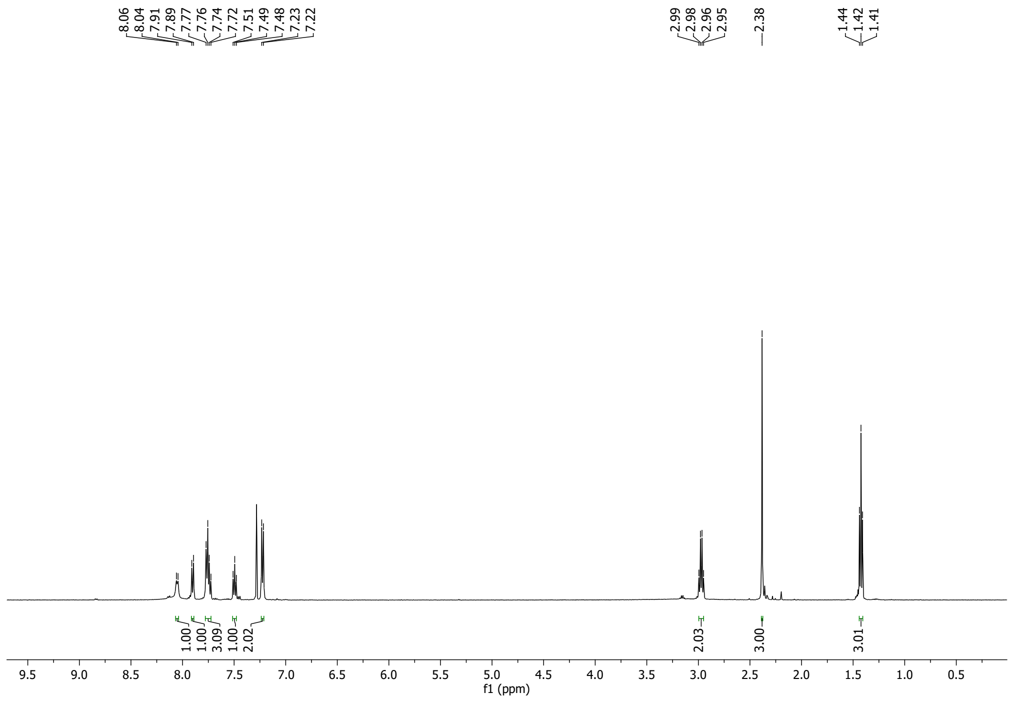

^{13}C NMR of compound **3k**

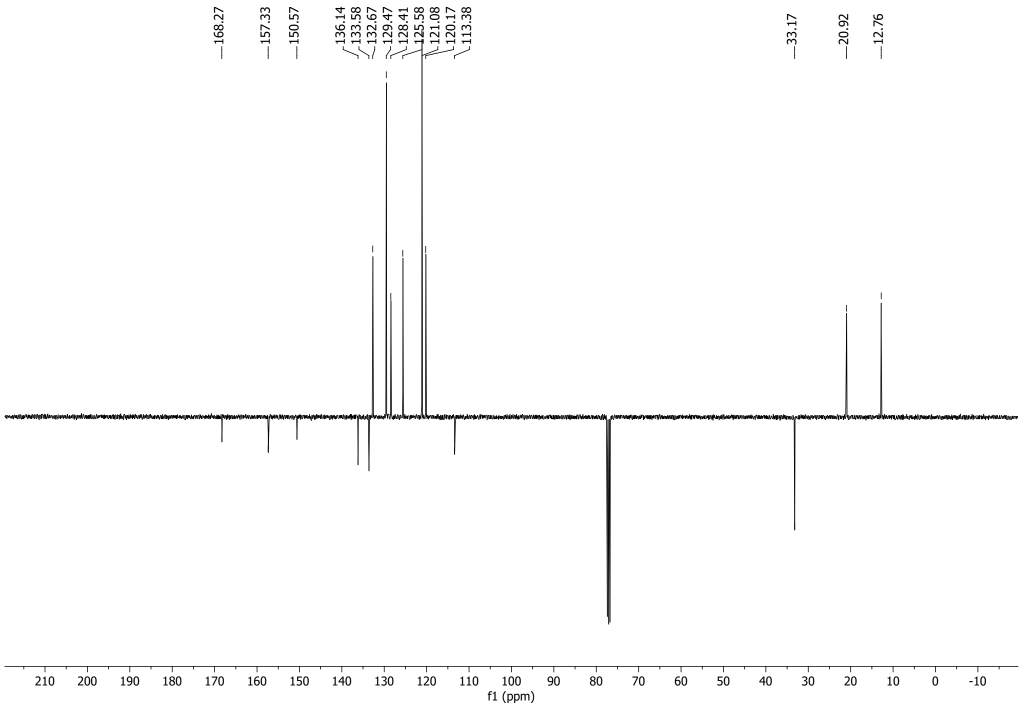

Figure S12. ^{1}H NMR and ^{13}C NMR of 4-(2,3-Dimethylphenyl)aminoquinazoline 3l

^{1}H NMR of compound **3l**

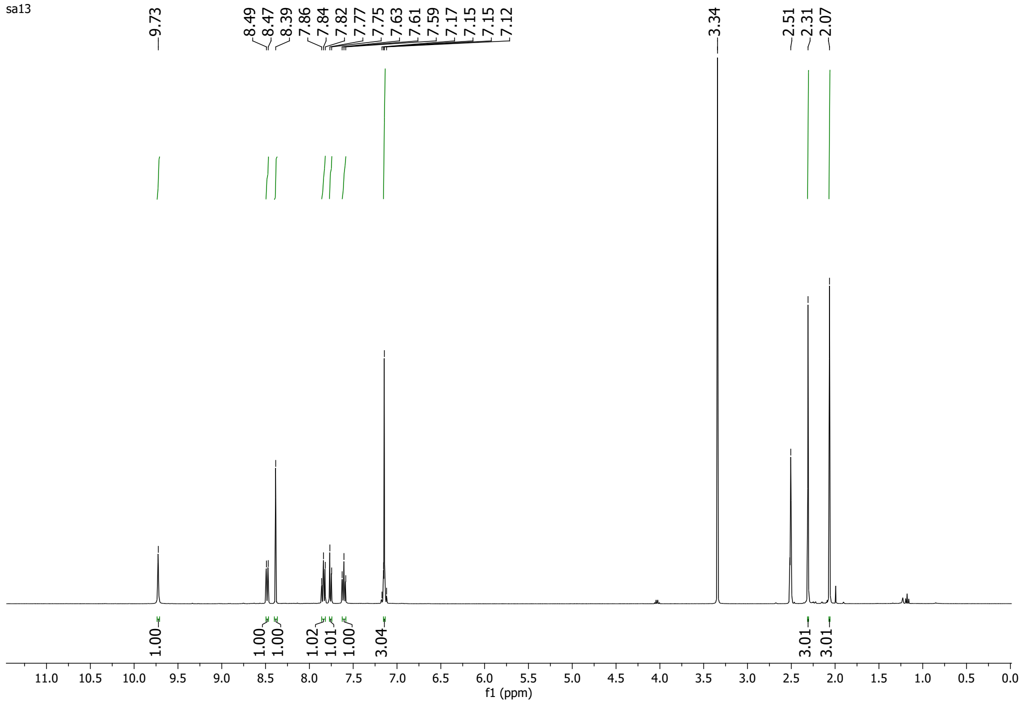

^{13}C NMR of compound **3l**

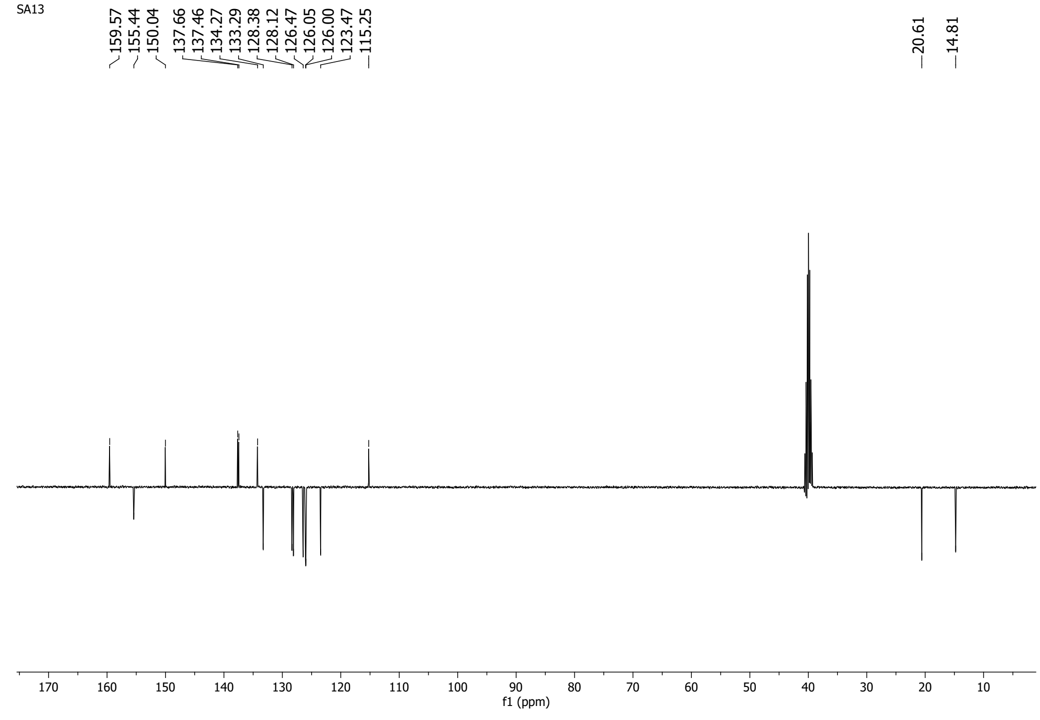

Figure S13. ^{1}H NMR and ^{13}C NMR of 2-methyl-4-(2,3-Dimethylphenyl)aminoquinazoline 3m

^{1}H NMR of compound **3m**

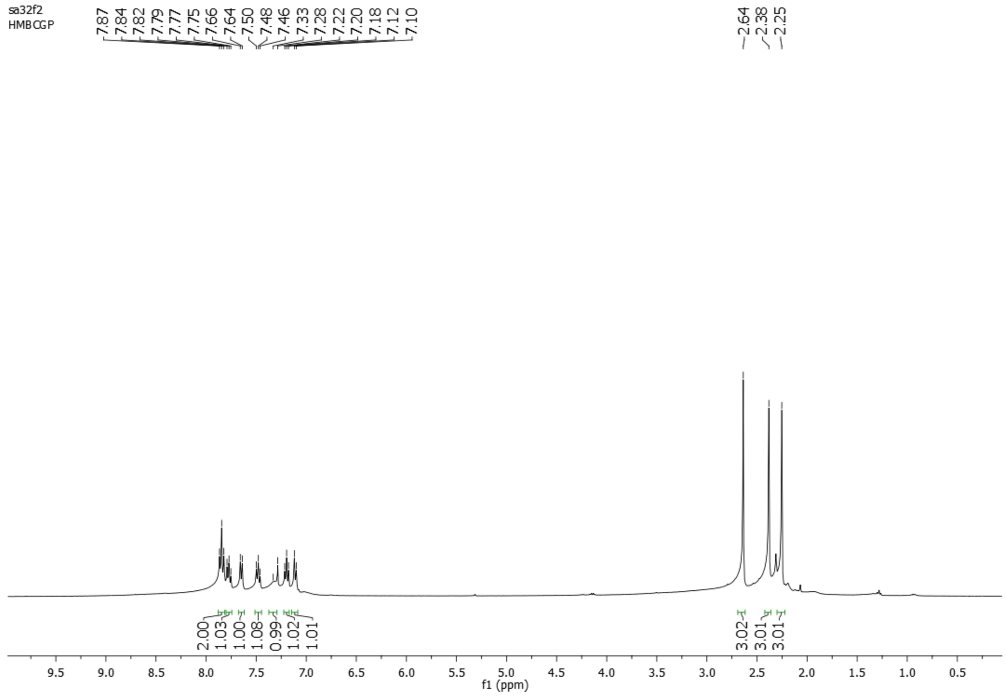

^{13}C NMR of compound **3m**

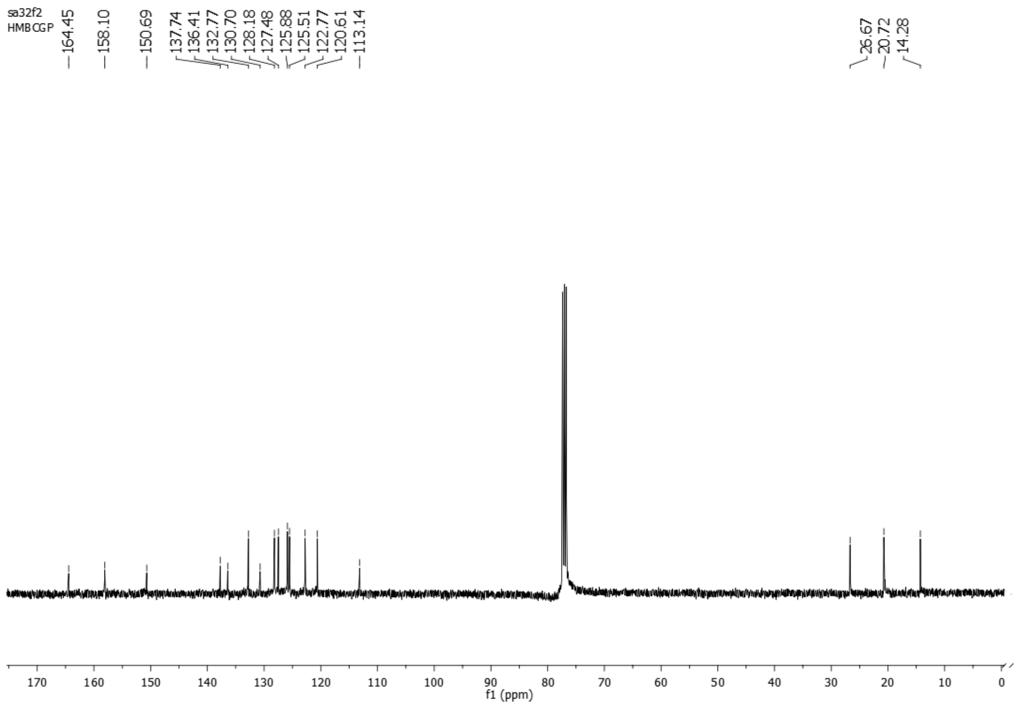

Figure S14. ^{1}H NMR and ^{13}C NMR of 4-(4-Nitrophenyl)aminoquinazoline 3n

^{1}H NMR of compound **3n**

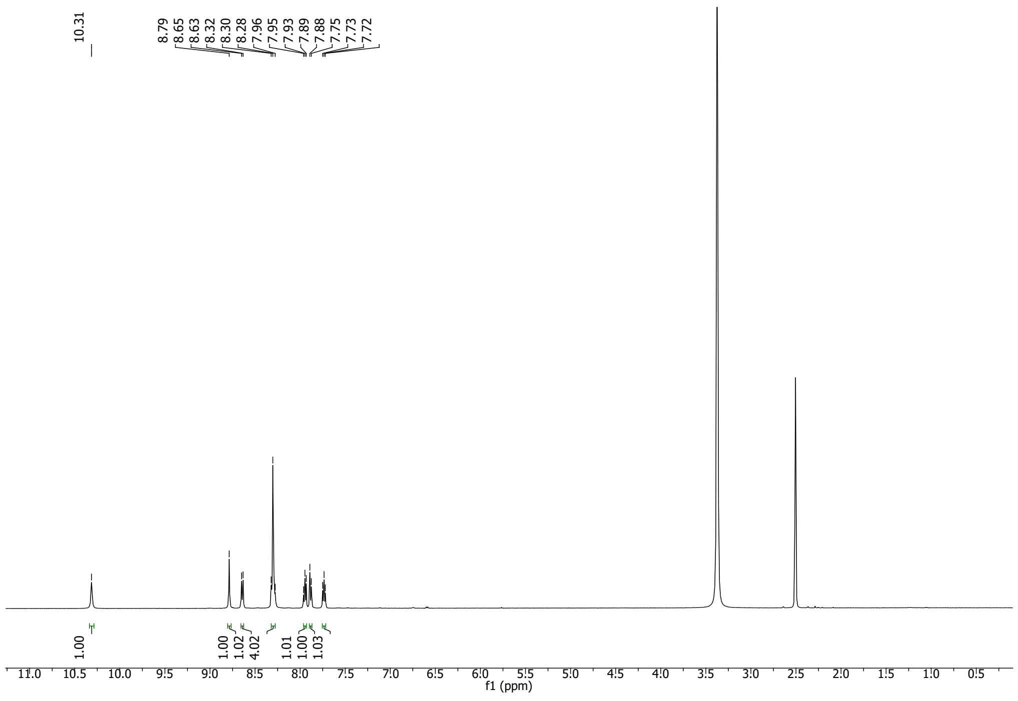

^{13}C NMR of compound **3n**

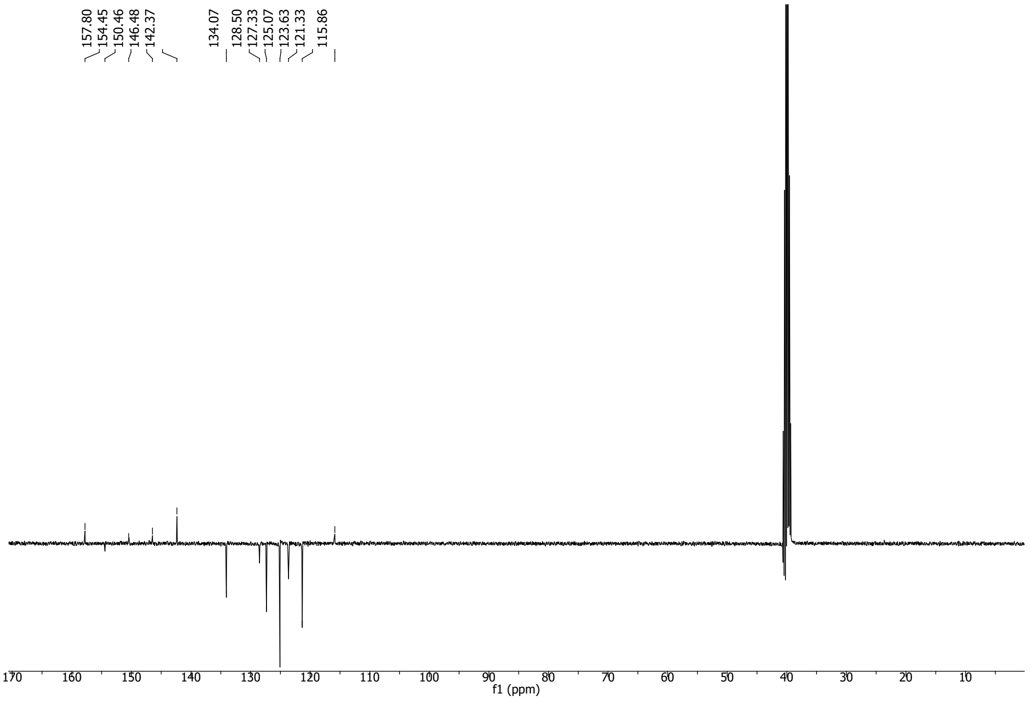

Figure S15. ^{1}H NMR and ^{13}C NMR of 2-Methyl-*4-(*4-nitrophenyl)aminoquinazoline 3o

^{1}H NMR of compound **3o**

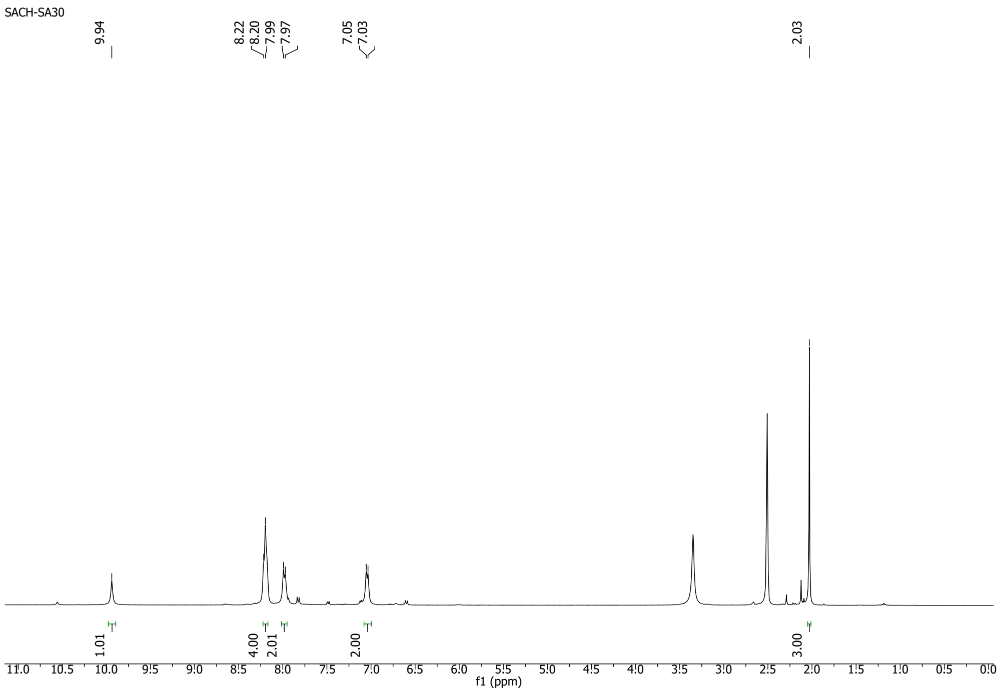

^{13}C NMR of compound **3o**

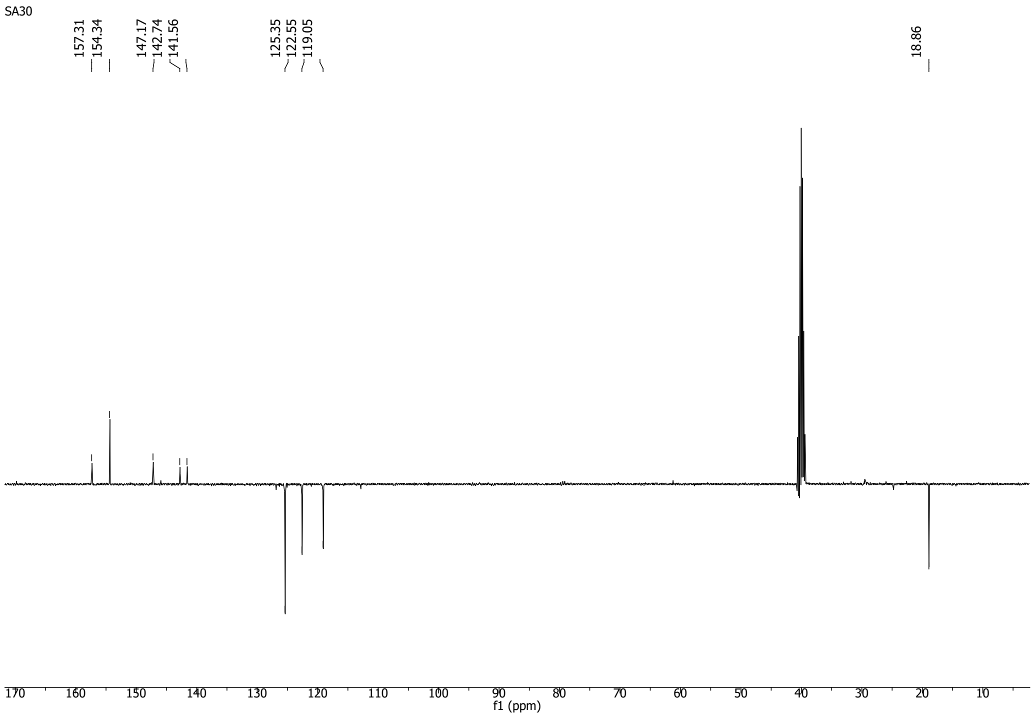

Figure S16. ^{1}H NMR and ^{13}C NMR of 4-(4-Chlorophenyl)aminoquinazoline 3p

^{1}H NMR of compound **3p**

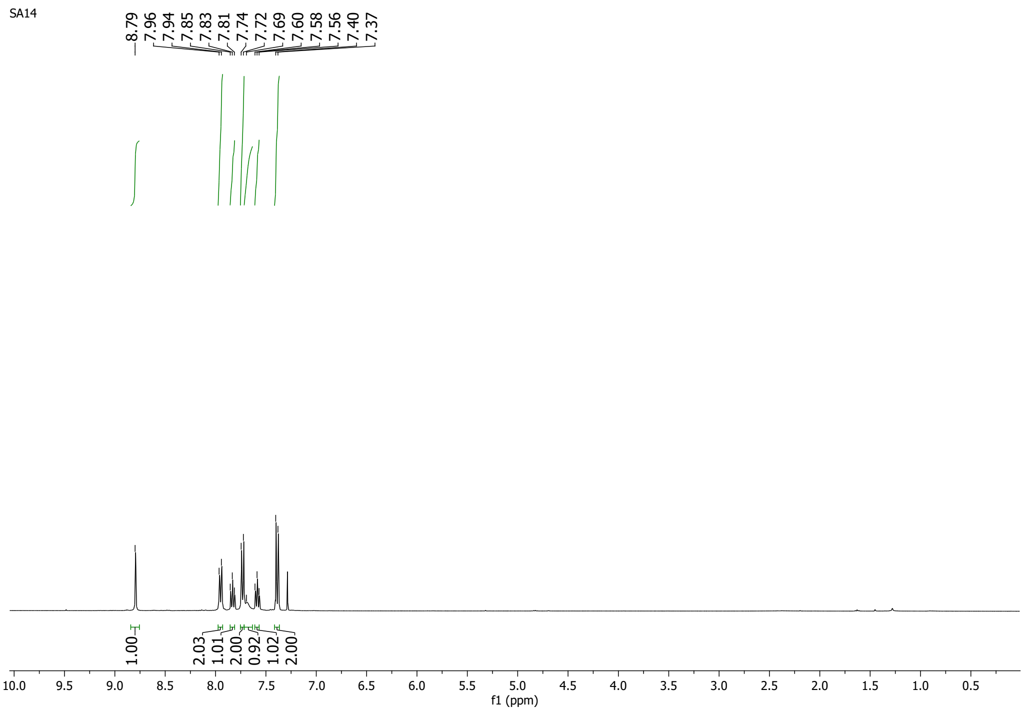

^{13}C NMR of compound **3p**

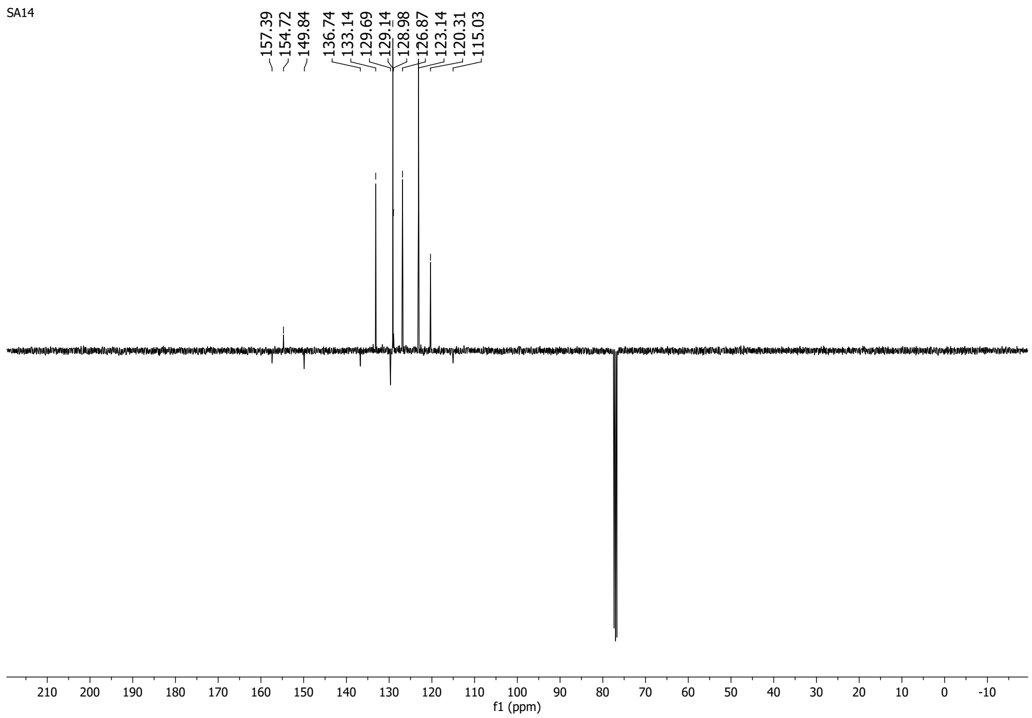

Figure S17. ^{1}H NMR and ^{13}C NMR of 2-ethyl-4-(4-Chlorophenyl)aminoquinazoline 3q

^{1}H NMR of compound **3q**

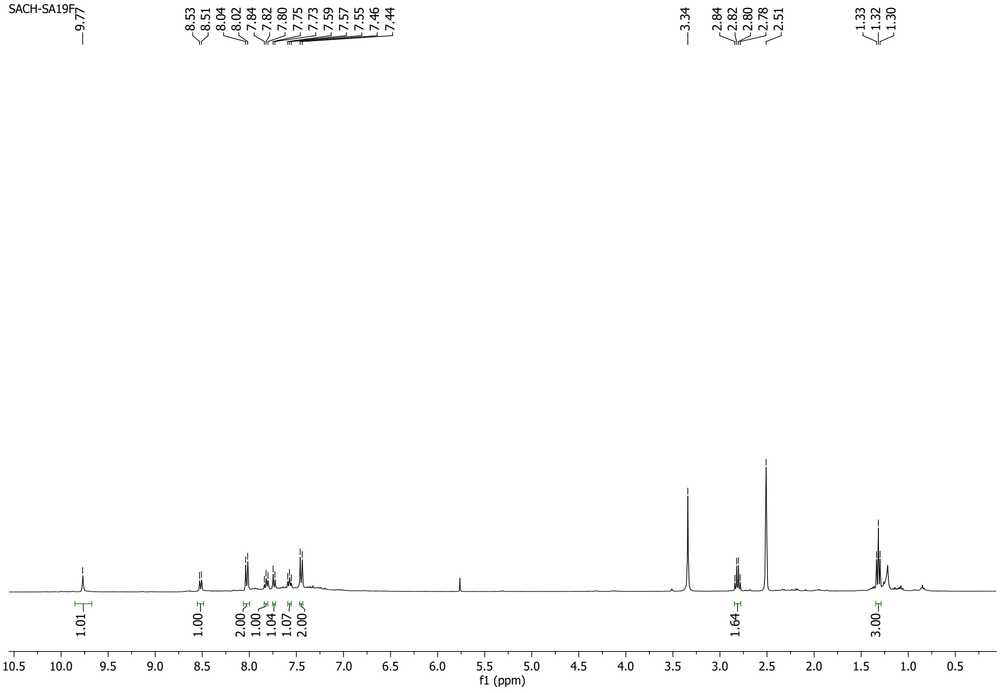

^{13}C NMR of compound **3q**

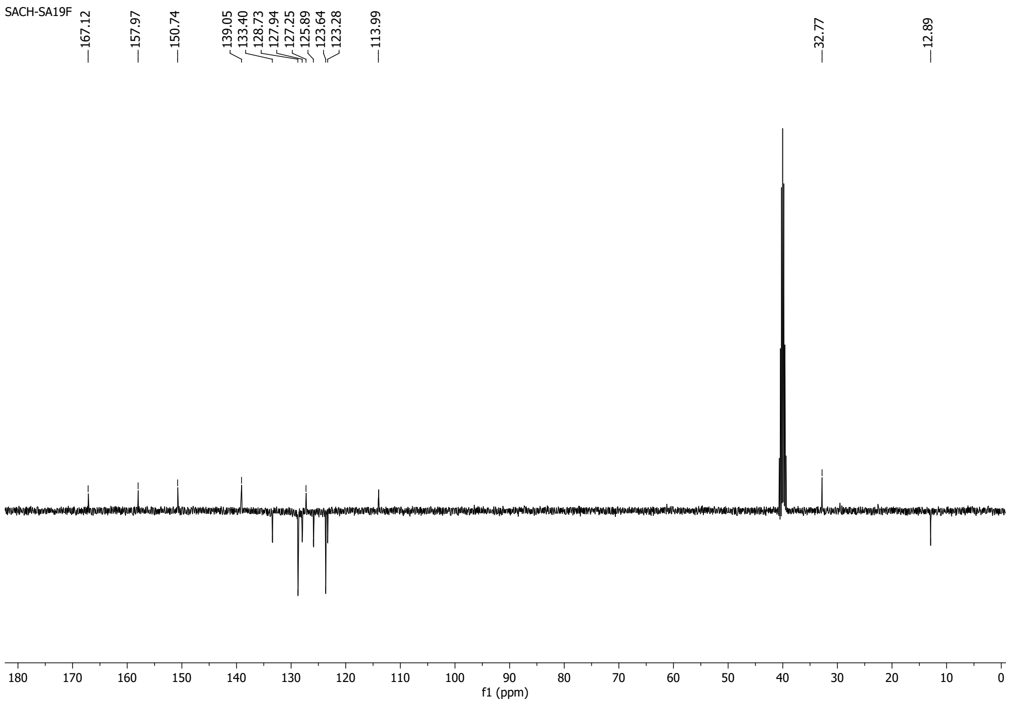

Figure S18. HSQC of compound **3a**

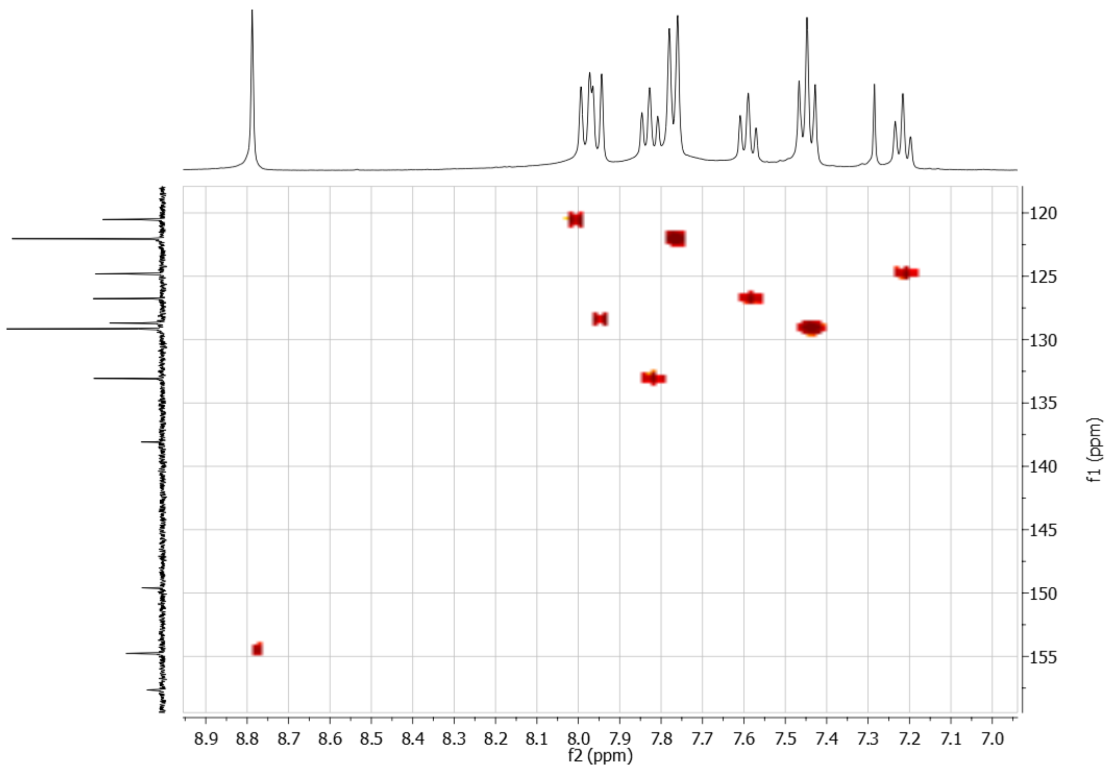

Figure S19. HMBC of compound **3a**

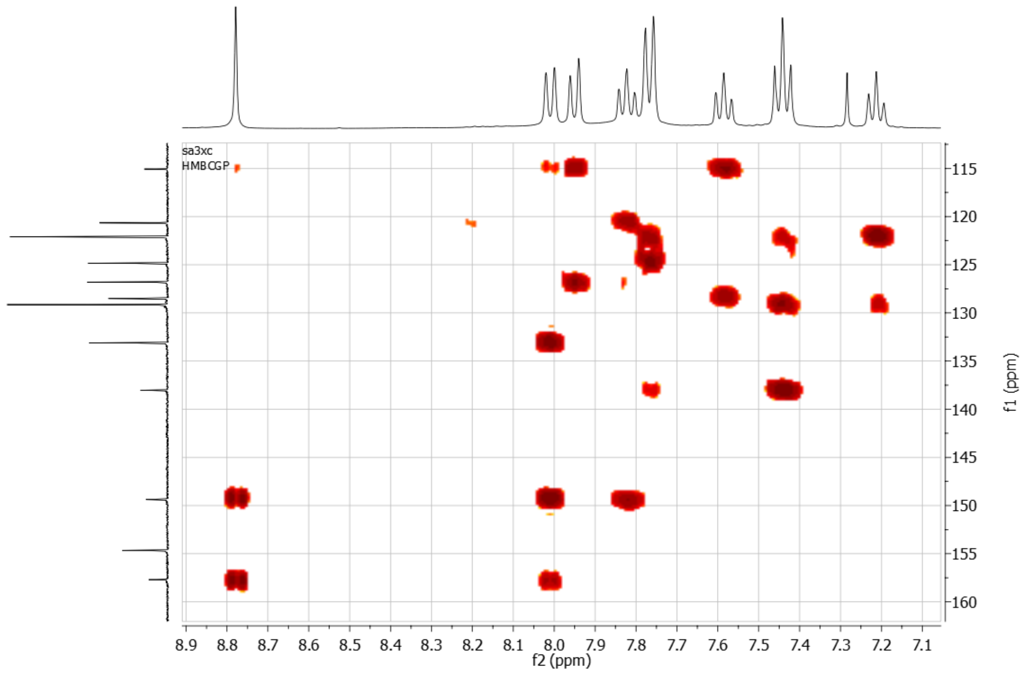

Figure S20. HSQC of compound **3d**

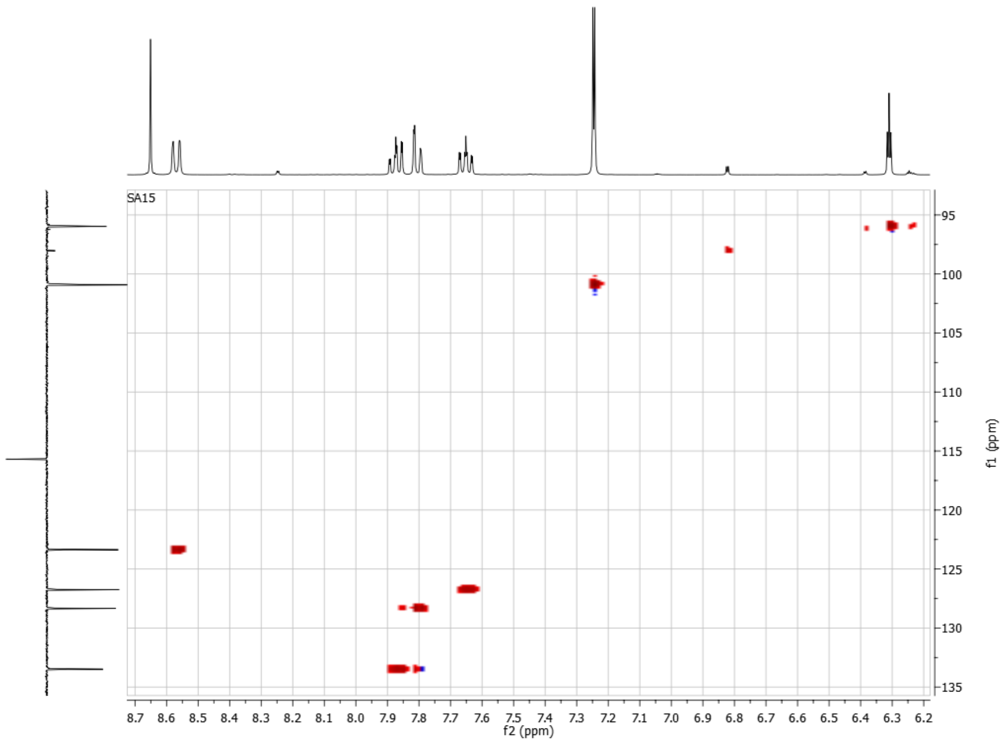

Figure S21. HMBC of compound **3d**

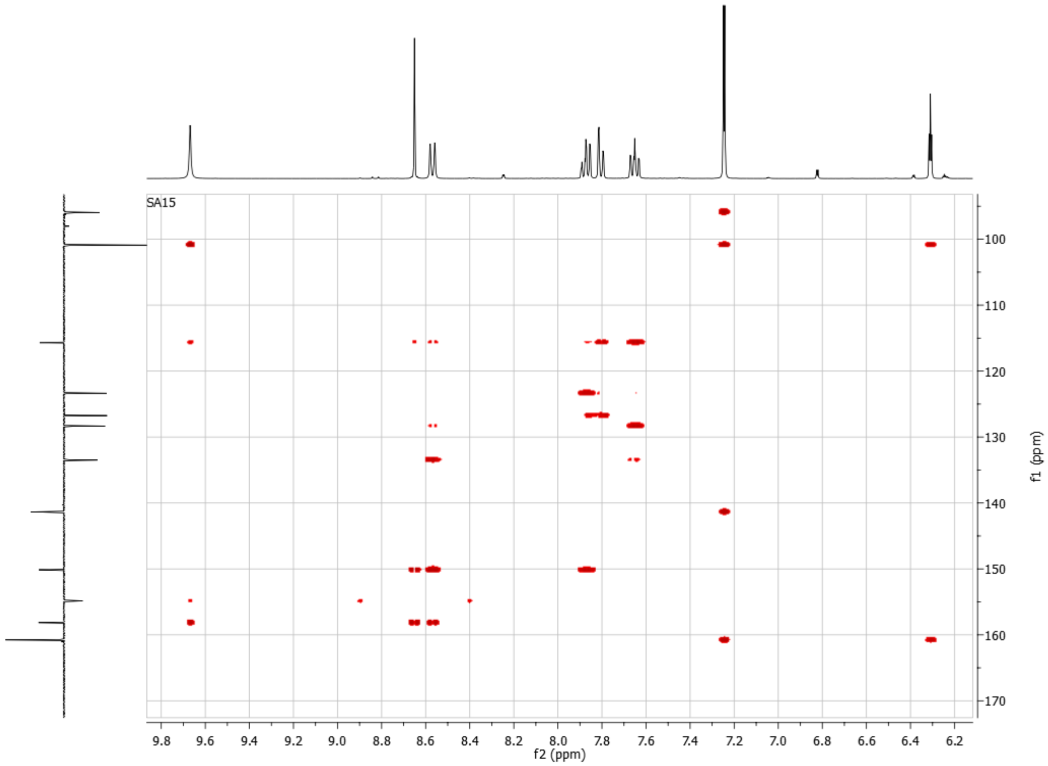

Figure S22. HRMS spectra of compound **3a**

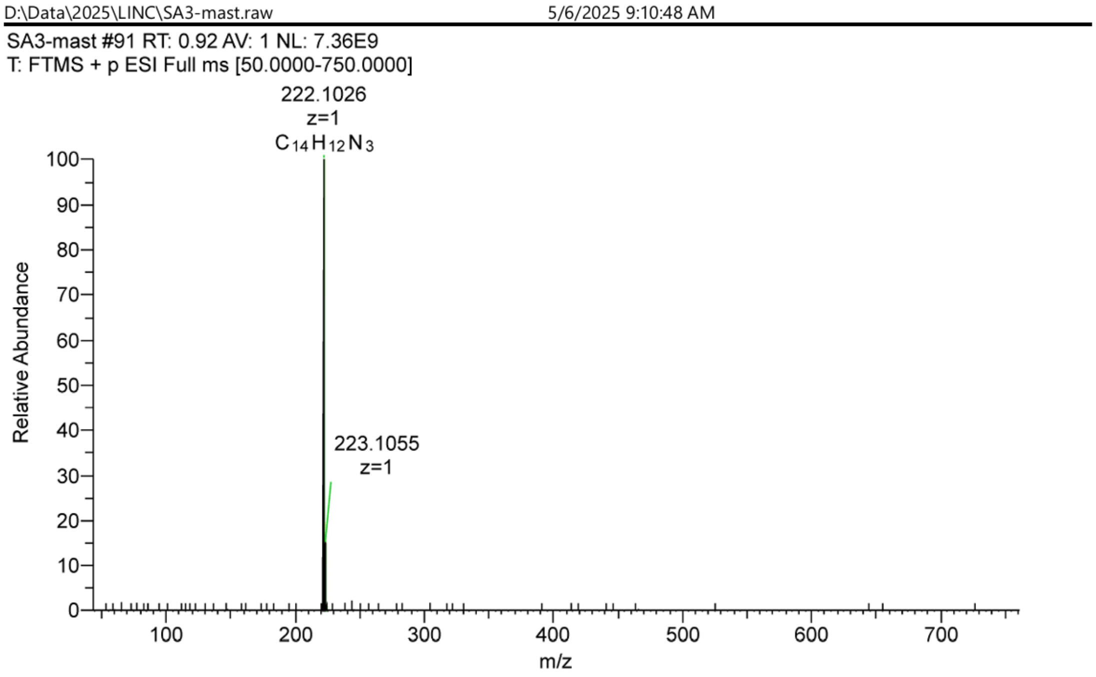

Peak Mass	Display Formula	Delta [ppm]	Theo. mass	Combined Score	Pattern Cov. [%]	MSMS Matched Fr...
222.1026	$C_{14}H_{12}N_3$	-0.02	222.10257	98.08	100	(Collection)

Figure S23. HRMS spectra of compound **3b**

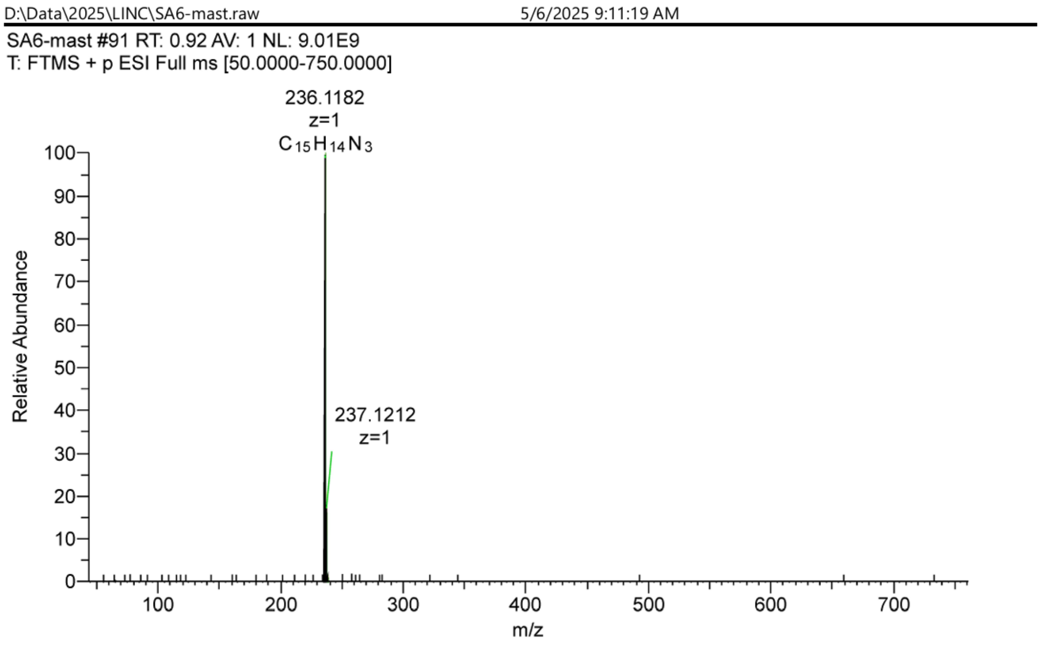

Peak Mass	Display Formula	Delta [ppm]	Theo. mass	Combined Score	Pattern Cov. [%]	MSMS Matched Fragments
236.1182	$C_{15}H_{14}N_3$	-0.25	236.11822	97.58	100	(Collection)

Figure S24. HRMS spectra of compound **3c**

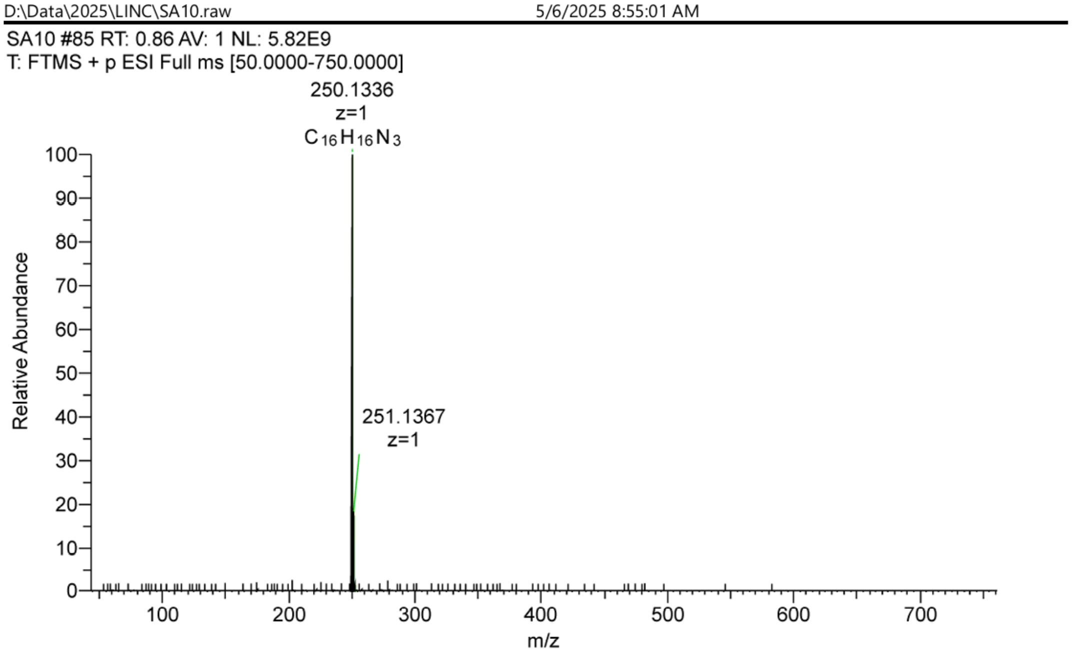

Peak Mass	Display Formula	Delta [ppm]	Theo. mass	Combined Score	Pattern Cov. [%]	MSMS Matched Fr...
250.1336	$C_{16}H_{16}N_3$	-1.13	250.13387	97.14	99.08	(Collection)

Figure S25. HRMS spectra of compound **3d**

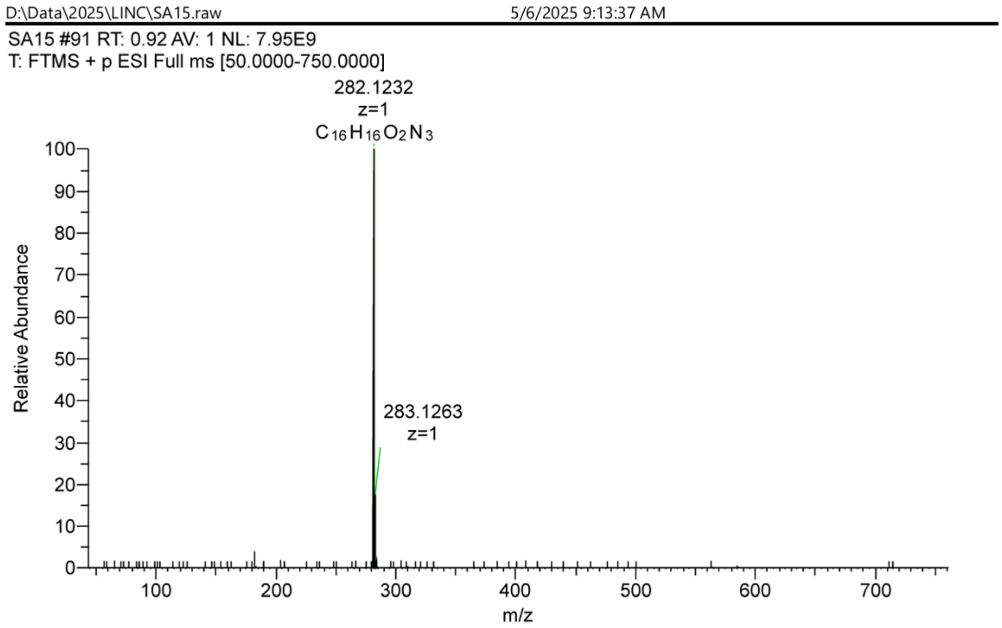

Peak Mass	Display Formula	Delta [ppm]	Theo. mass	Combined Score	Pattern Cov. [%]	MSMS Matched Fragments
282.1232	$C_{16}H_{16}O_2N_3$	-1.68	282.12370	97.64	100	(Collection)

Figure S26. HRMS spectra of compound **3e**

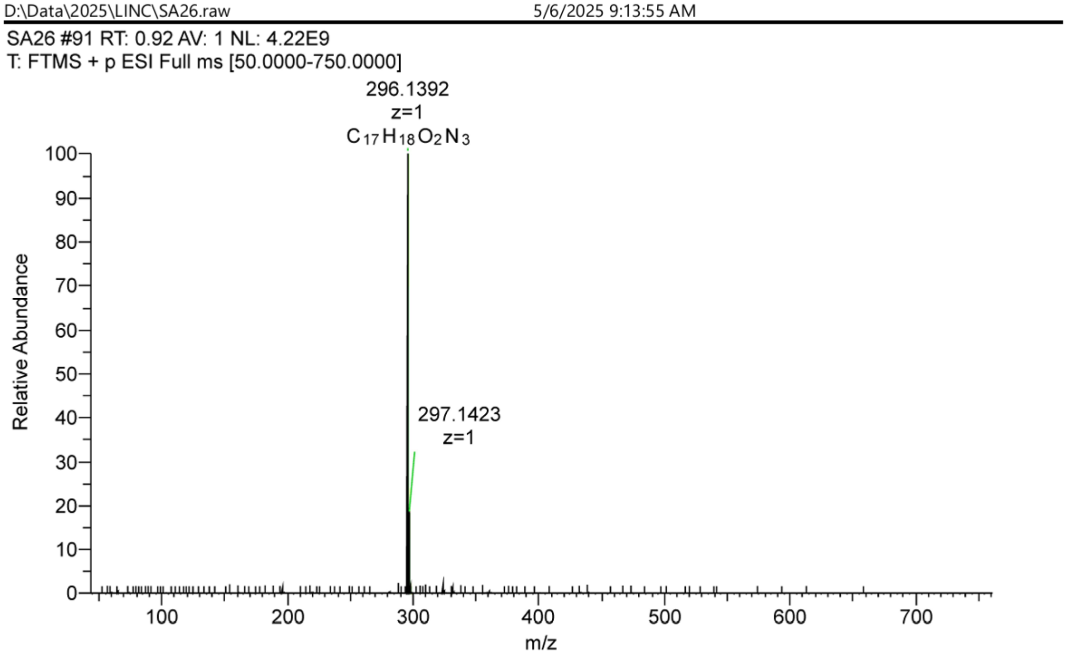

Peak Mass	Display Formula	Delta [ppm]	Theo. mass	Combined Score	Pattern Cov. [%]	MSMS Matched Fragments
296.1392	$C_{17}H_{18}O_2N_3$	-0.45	296.13935	97.02	99.89	(Collection)

Figure S27. HRMS spectra of compound **3f**

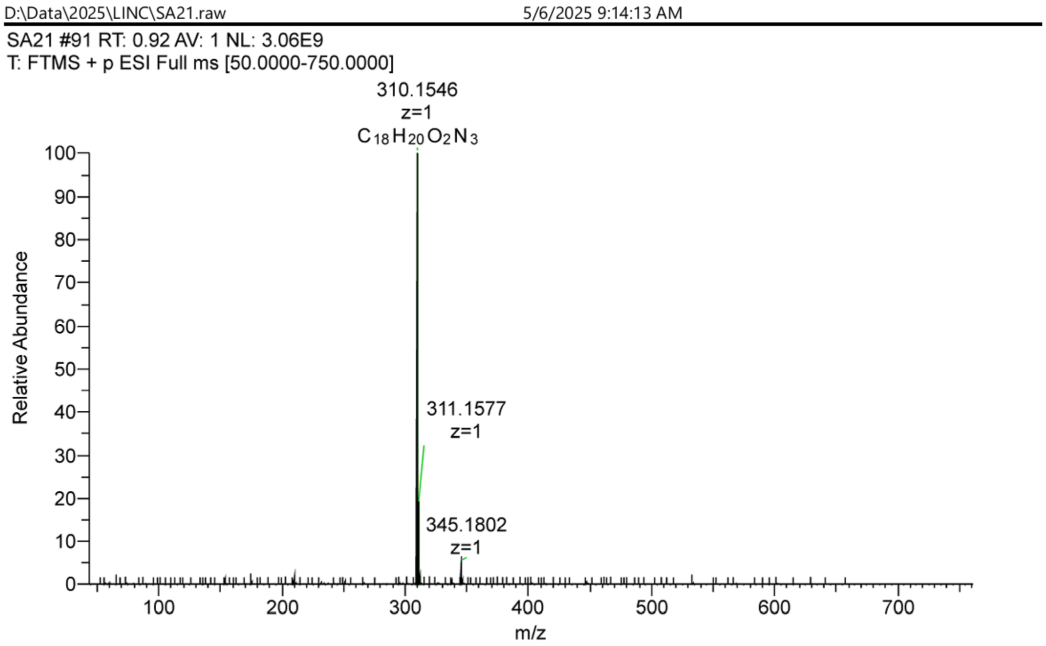

Peak Mass	Display Formula	Delta [ppm]	Theo. mass	Combined Score	Pattern Cov. [%]	MSMS Matched Fragments
310.1546	$C_{18}H_{20}O_2N_3$	-1.29	310.15500	97.92	100	(Collection)

Figure S28. HRMS spectra of compound **3g**

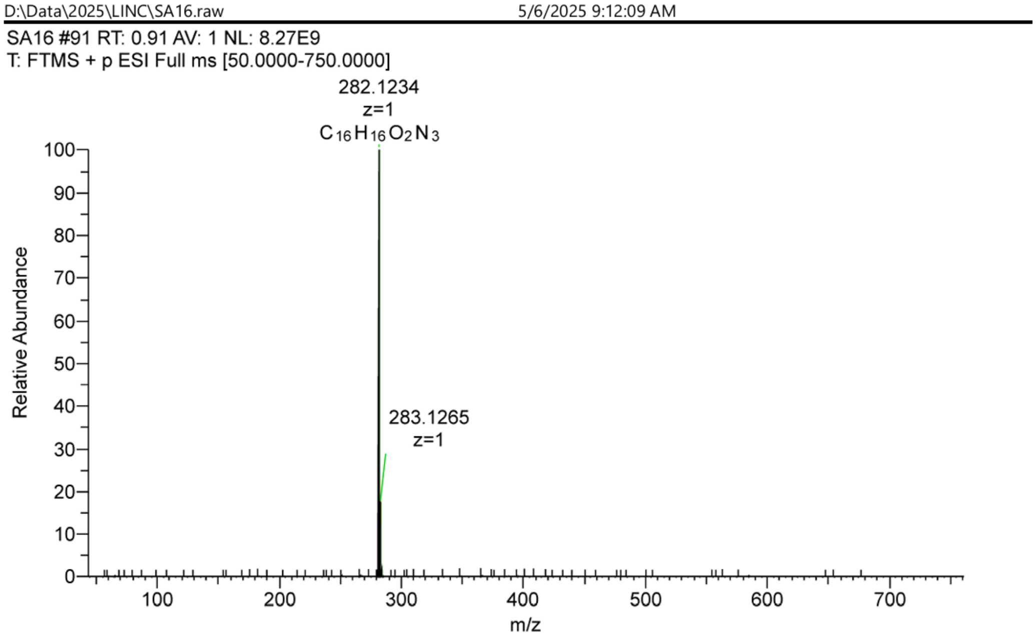

Peak Mass	Display Formula	Delta [ppm]	Theo. mass	Combined Score	Pattern Cov. [%]	MSMS Matched Fragments
282.1234	$C_{16}H_{16}O_2N_3$	-1.03	282.12370	98.59	100	(Collection)

Figure S29. HRMS spectra of compound **3h**

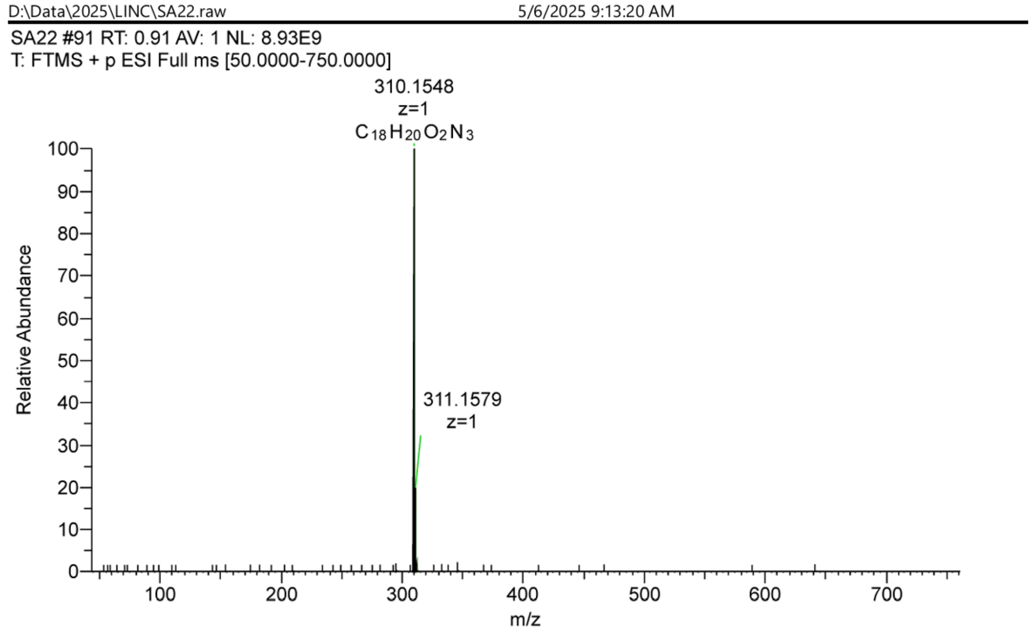

Peak Mass	Display Formula	Delta [ppm]	Theo. mass	Combined Score	Pattern Cov. [%]	MSMS Matched Fragments
310.1548	$C_{18}H_{20}O_2N_3$	-0.51	310.15500	97.08	99.87	(Collection)

Figure S30. HRMS spectra of compound **3i**

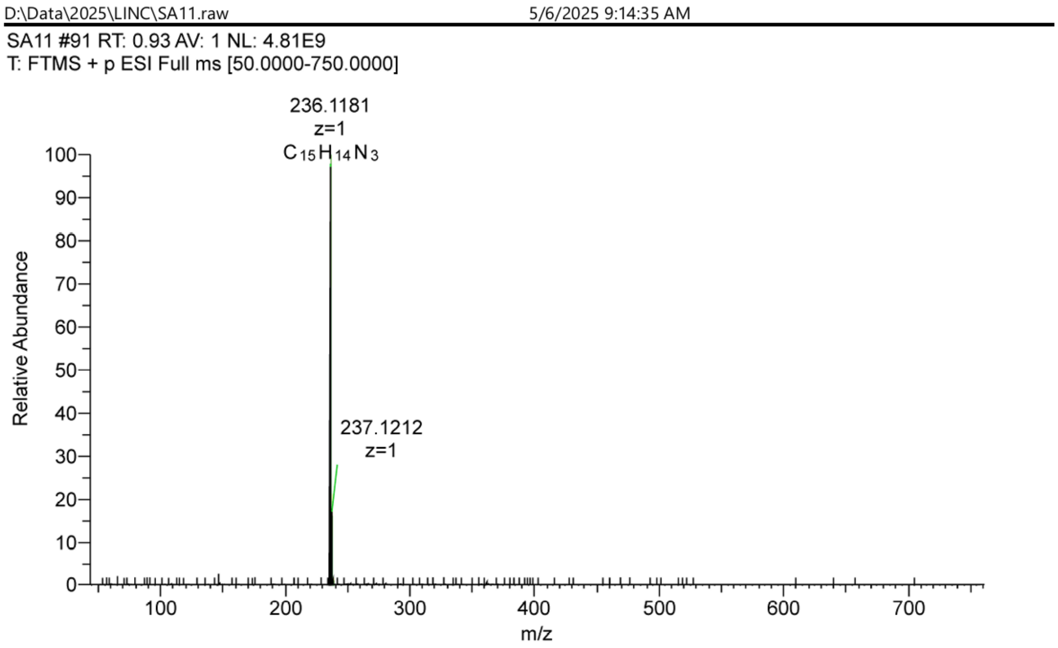

Peak Mass	Display Formula	Delta [ppm]	Theo. mass	Combined Score	Pattern Cov. [%]	MSMS Matched Fr...
236.1181	$C_{15}H_{14}N_3$	-0.38	236.11822	97.8	100	(Collection)

Figure S31. HRMS spectra of compound **3j**

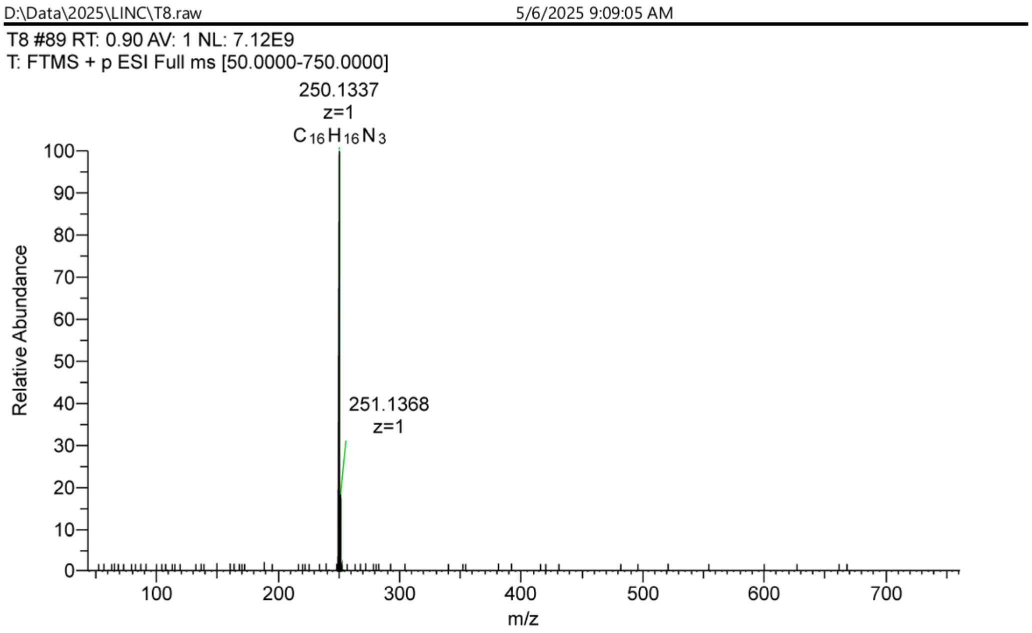

Peak Mass	Display Formula	Delta [ppm]	Theo. mass	Combined Score	Pattern Cov. [%]	MSMS Matched Fragments
250.1337	$C_{16}H_{16}N_3$	-0.52	250.13387	97.16	99.08	(Collection)

Figure S32. HRMS spectra of compound **3k**

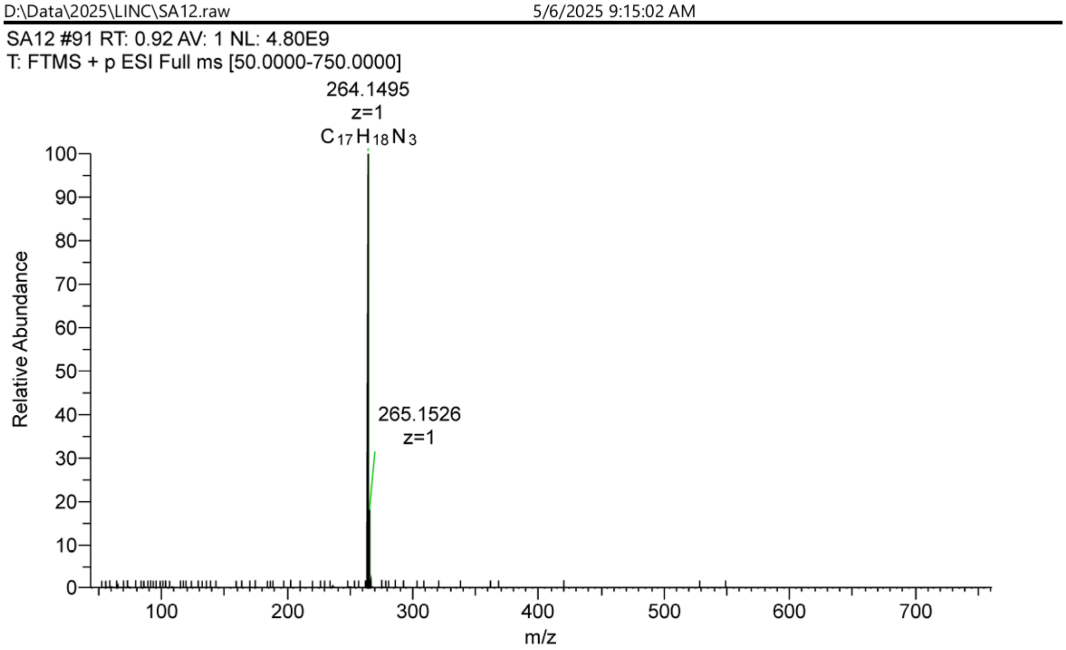

Peak Mass	Display Formula	Delta [ppm]	Theo. mass	Combined Score	Pattern Cov. [%]	MSMS Matched Fragments
264.1495	$C_{17}H_{18}N_3$	-0.19	264.14952	97.1	99.08	(Collection)

Figure S33. HRMS spectra of compound **3l**

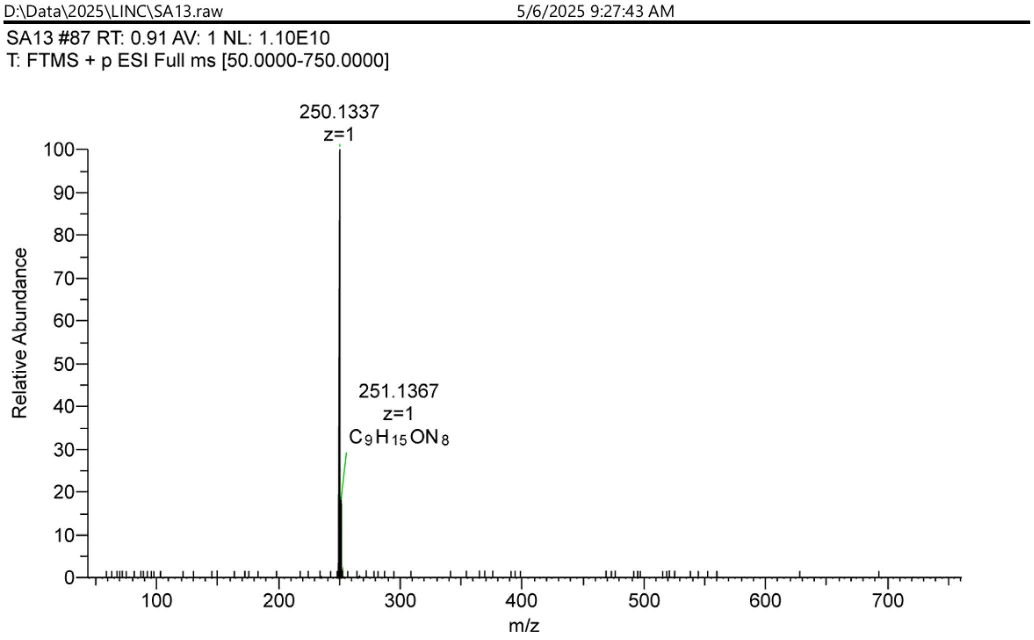

Peak Mass	Display Formula	Delta [ppm]	Theo. mass	Combined Score	Pattern Cov. [%]	MSMS Matched Fr...
251.1367	$C_9H_{15}ON_8$	1.65	251.13633	95.13	97.14	(Collection)

Figure S34. HRMS spectra of compound **3m**

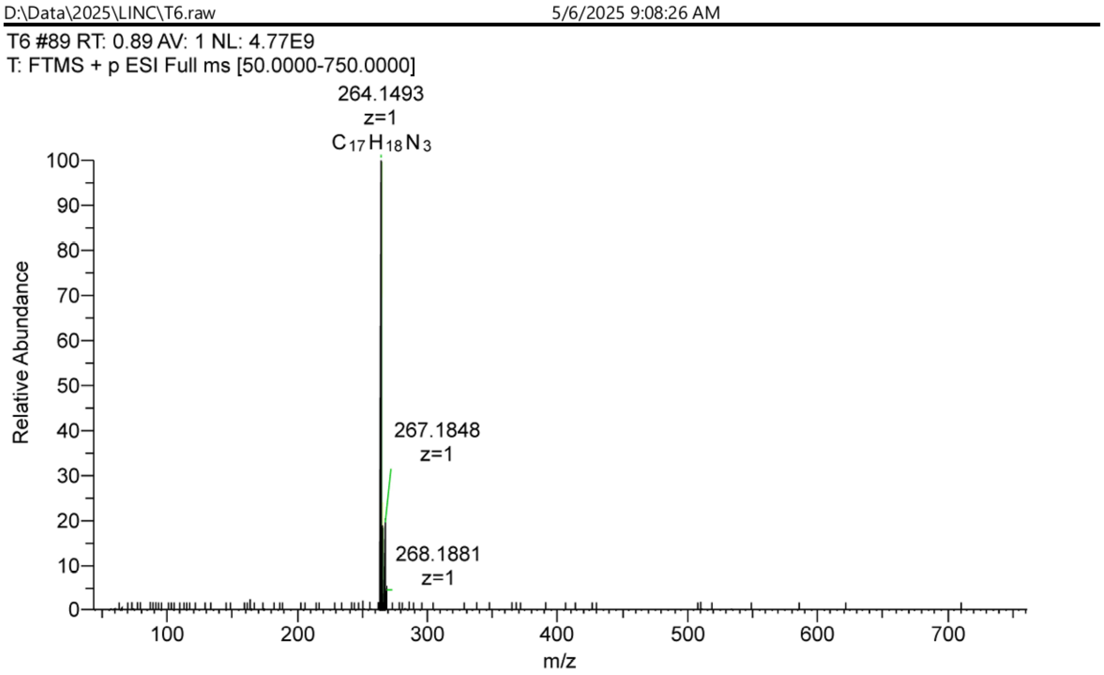

Peak Mass	Display Formula	Delta [ppm]	Theo. mass	Combined Score	Pattern Cov. [%]	MSMS Matched Fragments
264.1493	$C_{17}H_{18}N_3$	-0.76	264.14952	96.28	98.91	(Collection)

Figure S35. HRMS spectra of compound **3n**

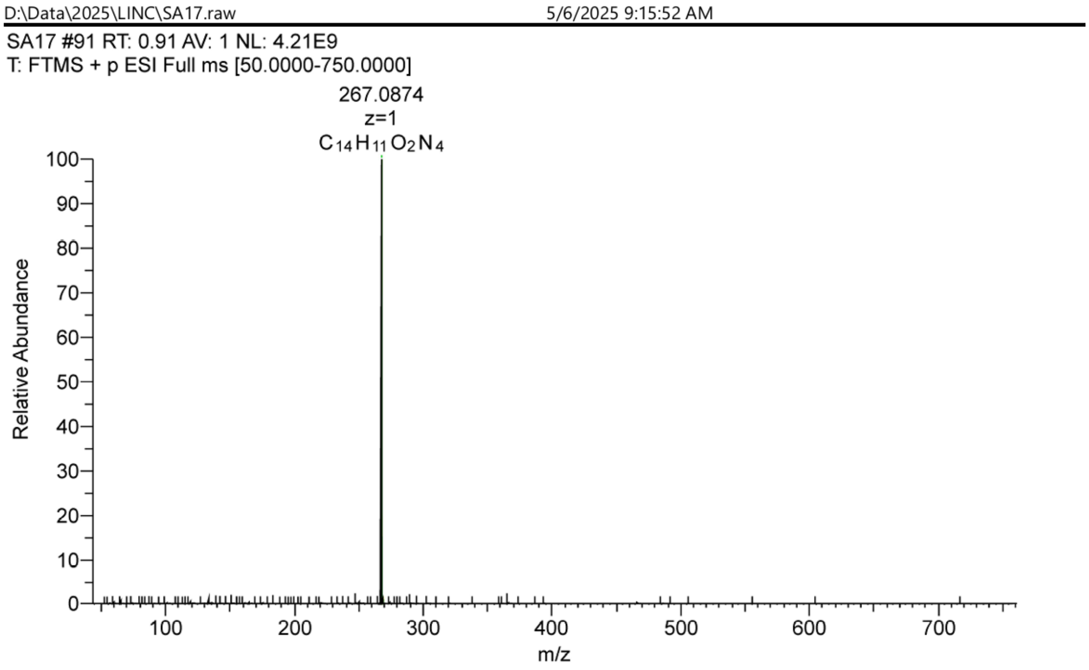

Peak Mass	Display Formula	Delta [ppm]	Theo. mass	Combined Score	Pattern Cov. [%]	MSMS Matched Fragments
267.0874	$C_{14}H_{11}O_2N_4$	-0.82	267.08765	96.96	98.74	(Collection)

Figure S36. HRMS spectra of compound **3o**

D:\Data\2025\LINC\SA30.raw 5/6/2025 9:18:09 AM

SA30 #91 RT: 0.92 AV: 1 NL: 1.95E8
T: FTMS + p ESI Full ms [50.0000-750.0000]

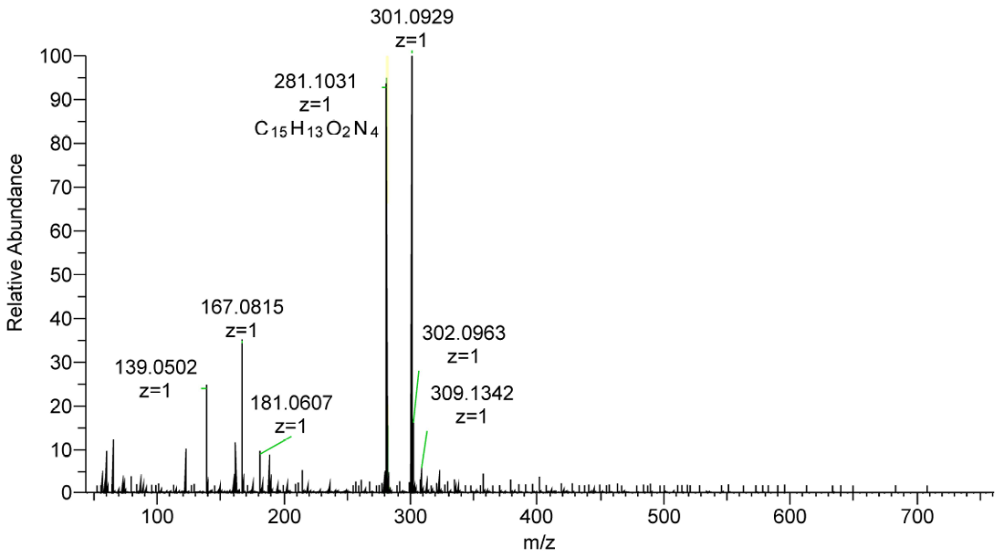

Peak Mass	Display Formula	Delta [ppm]	Theo. mass	Combined Score	Pattern Cov. [%]	MSMS Matched Fragments
281.1031	$C_{15}H_{13}O_2N_4$	-0.65	281.10330	92.99	98.74	(Collection)

Figure S37. HRMS spectra of compound **3p**

D:\Data\2025\LINC\SA14.raw 5/6/2025 9:06:25 AM

SA14 #89 RT: 0.89 AV: 1 NL: 5.56E9
T: FTMS + p ESI Full ms [50.0000-750.0000]

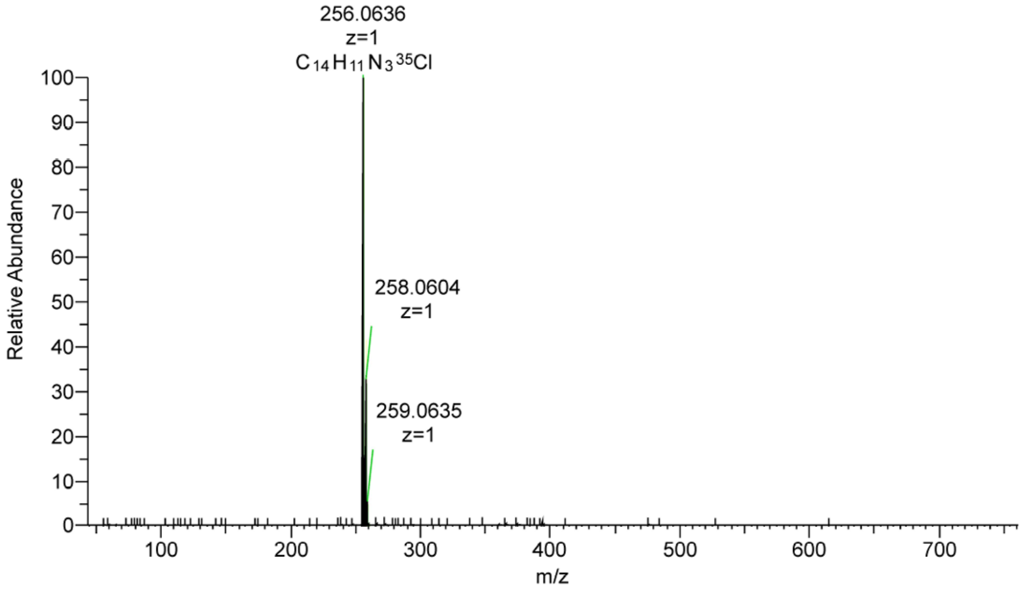

Peak Mass	Display Formula	Delta [ppm]	Theo. mass	Combined Score	Pattern Cov. [%]	MSMS Matched Fr...
256.0636	$C_{14}H_{11}N_3{}^{35}Cl$	-0.13	256.06360	97.48	99.31	(Collection)

Figure S38. HRMS spectra of compound **3q**

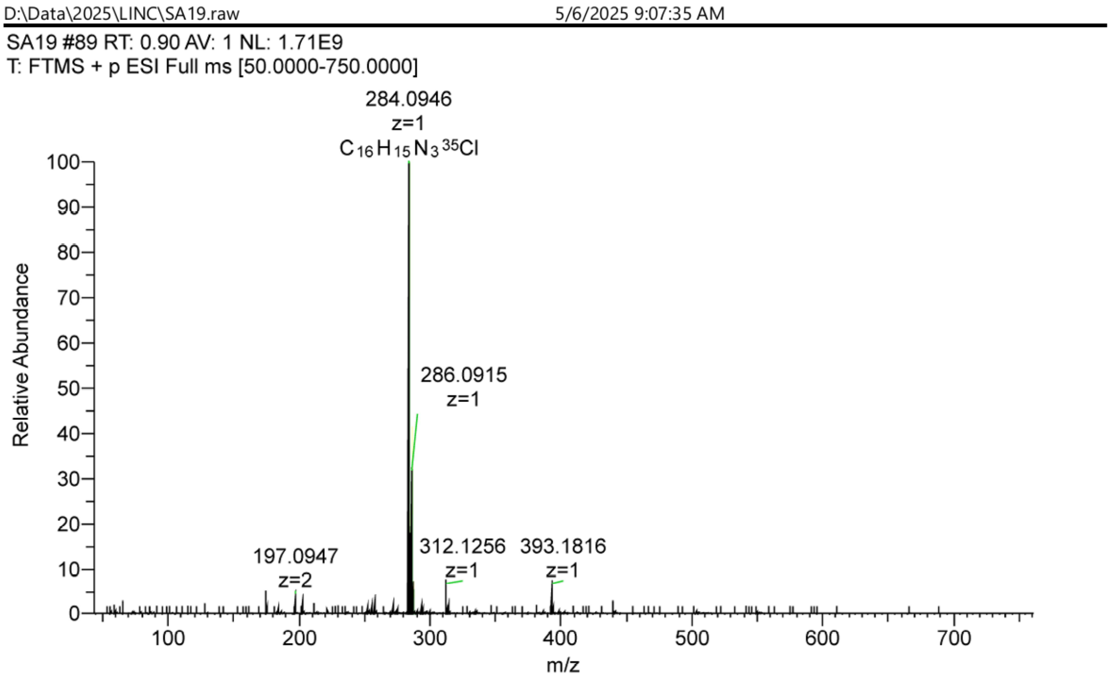

Peak Mass	Display Formula	Delta [ppm]	Theo. mass	Combined Score	Pattern Cov. [%]	MSMS Matched Fragments
284.0946	$C_{16}H_{15}N_3{}^{35}Cl$	-0.94	284.09490	96.93	99.1	(Collection)
